# Insertion of Phosphenium Ions into a Bicyclo[1.1.0]Tetraphosphabutane Iron Complex

**DOI:** 10.3390/molecules26133920

**Published:** 2021-06-26

**Authors:** Martin Weber, Gábor Balázs, Alexander V. Virovets, Eugenia Peresypkina, Manfred Scheer

**Affiliations:** Institute of Inorganic Chemistry, University of Regensburg, Universitätsstrasse 31, 93040 Regensburg, Germany; Martin2.Weber@chemie.uni-regensburg.de (M.W.); gabor.balazs@chemie.uni-regensburg.de (G.B.); avvirovets@yahoo.com (A.V.V.); eugeniaperesypkina@gmail.com (E.P.)

**Keywords:** bicyclo[1.1.0]tetraphosphabutane, phosphorus activation, arsenium ions, phosphenium ions, tetracyclo[3.3.0.0^2,7^.0^3,6^]octaphosphaoctane, P_4_ butterfly complex, R_2_P^+^ insertion

## Abstract

By reacting [{Cp‴Fe(CO)_2_}_2_(µ,η^1:1^-P_4_)] (**1**) with in situ generated phosphenium ions [Ph_2_P][A] ([A]^−^ = [OTf]^−^ = [O_3_SCF_3_]^−^, [PF_6_]^−^), a mixture of two main products of the composition [{Cp‴Fe(CO)_2_}_2_(µ,η^1:1^-P_5_(C_6_H_5_)_2_)][PF_6_] (**2a** and **3a**) could be identified by extensive ^31^P NMR spectroscopic studies at 193 K. Compound **3a** was also characterized by X-ray diffraction analysis, showing the rarely observed bicyclo[2.1.0]pentaphosphapentane unit. At room temperature, the novel compound [{Cp‴Fe}(µ,η^4:1^-P_5_Ph_2_){Cp‴(CO)_2_Fe}][PF_6_] (**4**) is formed by decarbonylation. Reacting **1** with in situ generated diphenyl arsenium ions gives short-lived intermediates at 193 K which disproportionate at room temperature into tetraphenyldiarsine and [{Cp‴Fe(CO)_2_}_4_(µ_4_,η^1:1:1:1^-P_8_)][OTf]_2_ (**5**) containing a tetracyclo[3.3.0.0^2,7^.0^3,6^]octaphosphaoctane ligand.

## 1. Introduction

**Scheme 1:**
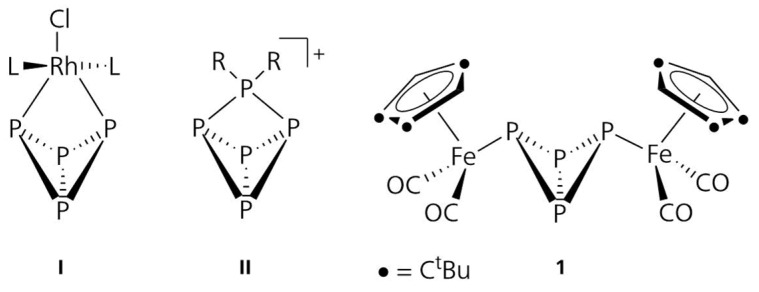
Selected compounds with a tetraphosphabicyclo[1.1.0] unit [6,7,16].

The activation of small molecules such as N_2_ or P_4_ with reactive transition metal fragments or main group reactants is one of the important aspects of modern chemistry. More energy-efficient reaction pathways leading, by higher atom economy, to compounds containing nitrogen or phosphorus seem more tangible with every new insight gained into the activation pathways [1,2,3,4,5]. The opening of one P-P bond of the P_4_ tetrahedron leading to the tetraphosphabicyclo[1.1.0] unit (also called the butterfly unit) is one of the first activation steps for P_4_ and is well-studied for reactive transition metal compounds. The activation leads to a cage-like compound by insertion of a metal fragment as observed for example in [L_2_RhCl(η^1:1^-P_4_)] (L = PPh_3_, P(m-tol)_3_, P(p-tol)_3_, AsPh_3_) (**I**), the first reported polyphosphorus ligand complex (Scheme 1) [6]. Also, a µ,η^1:1^-coordination mode can be observed, e.g., in the complex [{Cp‴Fe(CO)_2_}_2_(µ,η^1:1^-P_4_)] (Cp‴ = C_5_H_2_(C(CH_3_)_3_)_3_) (**1**), synthesized by the reaction of [Cp‴Fe(CO)_2_]_2_ with white phosphorus [7,8]. Since the highest occupied molecular orbital (HOMO) in **1** is localized on the "wing tip" phosphorus atoms of the butterfly unit, we have investigated the reactivity towards Lewis-acidic transition metal fragments [9,10,11,12] or the coinage metal salts such as Cu(I), Ag(I) and Au(I) [11,12,13]. In most of the cases, the coordination of the "wing tip" phosphorus atoms of the intact butterfly to the electron-poor metal centre is observed, but also a rearrangement to a *cyclo*-P_4_ unit. The complex [{Cp*Cr(CO)_3_}_2_(µ,η^1:1^-P_4_)] (Cp* = C_5_(CH_3_)_5_), which is similar to **1**, isomerizes in reactions with M(CO)_5_ (M = Cr, Mo) entities, while fragmentation occurs in reactions with N-heterocyclic carbenes [13,14]. Beside transition metal compounds, P_4_ can be activated by main group electrophiles [2,15]. A prominent example for main group electrophiles which have been used in the activation of P_4_ are phosphenium cations [R_2_P]^+^ (R = alkyl or aryl) which, in general, are too reactive to be isolated, but can be generated in situ by halogen abstraction from halogenophosphines with abstractors, such as, for instance GaCl_3_, silver or thallium salts in the presence of a suitable substrate to react with.

That way, new cationic polyphosphorus compounds with catena [17,18,19,20,21,22], ring [22,23,24] or cage [16,22,25] units could be synthesized, in particular by Burford and Weigand, who observed the stepwise opening of the P-P bonds of the P_4_ tetrahedron by insertion of [R_2_P]^+^ in a solvent-free reaction set up to the cationic cage compounds [Ph_2_P_5_]^+^ (**II**), [Ph_4_P_6_]^2+^ and [Ph_6_P_7_]^3+^, respectively [16]. Besides P_4_, the tetrahedral complex [Cp‴Ni(η^3^-P_3_)] also reacts with phosphenium cations by insertion into one P-P bond [26]. In contrast, [Cp*Fe(η^5^-P_5_)] reacts with main group electrophiles, such as, for instance, H^+^ or CH_3_^+^ with the formation of a P-E bond [27]. Our preliminary studies have shown that **1** reacts with HBF_4_, leading to the protonation of one of the wing tip phosphorus atoms [12]. Therefore, the question arises as to whether the reaction of **1** with phosphenium cations leads to a simple coordination as observed for some transition metal fragments leading to type **I** complexes or to the insertion into one of the P-P bonds as reported for white phosphorus or [Cp‴Ni(η^3^-P_3_)], respectively. Furthermore, cations of the type [R_2_E]^+^ are not only known for phosphorus but also for the heavier homologue arsenic [28,29,30], yet no studies on the reactivity towards polyphosphorus compounds or white phosphorus are known. Herein, we report on the reactivity of **1** towards in situ generated phosphenium and arsenium cations [R_2_E]^+^ (E = P, As) leading to short-lived insertion and coordination products at low temperatures, which react further at room temperature to a *cyclo*-P_5_ unit in the case of E = P and to a compound featuring the rarely observed tetracyclo[3.3.0.0^2,7^.0^3,6^]octaphosphaoctane entity in the case of E = As.

## 2. Results

### 2.1. Reactions of **1** with [Ph_2_P]^+^[A]^−^

**Scheme 2:**
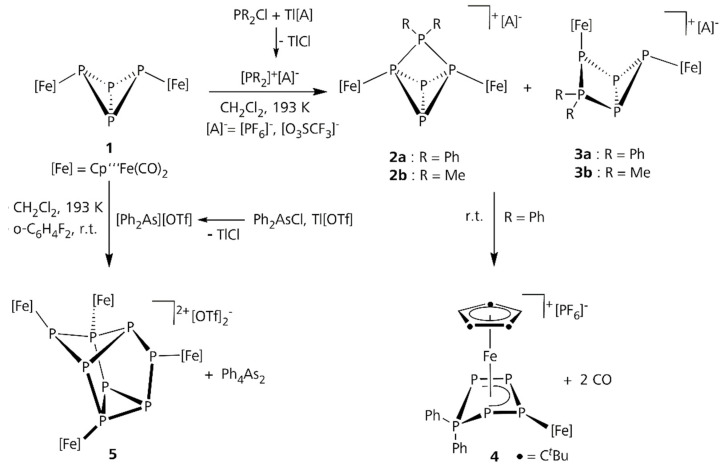
Reaction of **1** with in situ generated phosphenium and arsenium ions.

**Figure 1:**
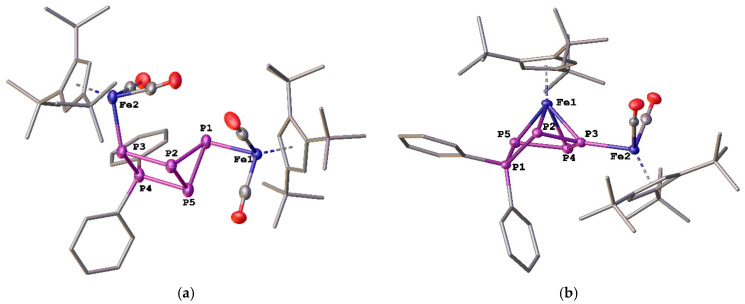
Cationic parts of the molecular structures of **3a** (**a**) and **4** (**b**), respectively, in the solid state. Hydrogen atoms, solvent molecules and counter ions are omitted for clarity. Selected bond lengths (Å) for **3a**—Fe1-P1 2.3162(12), Fe2-P3 2.3004(15), P1-P5 2.2010(18), P2-P1 2.1971(15), P2-P5 2.2546(17), P3-P2 2.2305(15), P3-P4 2.2128(17), P4-P5 2.1974(16). Selected bond lengths (Å) for **4**—Fe1-P3 2.2925(7), Fe1-P5 2.3422(7), Fe1-P4 2.3517(8), Fe1-P2 2.3610(8), Fe2-P3 2.2567(7), P1-P52.1590(9), P1-P2 2.1761(9), P2-P3 2.1293(9), P3-P4 2.1337(9), P4-P5 2.1322(10).

The complex [{Cp‴Fe(CO)_2_}_2_(µ,η^1:1^-P_4_)] (**1**) readily reacts with in situ generated diphenyl phosphenium salts generated from Ph_2_PCl and Tl[A] ([A]^−^ = [OTf]^−^ = [O_3_SCF_3_]^−^, [PF_6_]^−^) in ortho-difluorobenzene (o-DFB) at room temperature. In the ^31^P{^1^H} NMR spectrum of the reaction solution of [{Cp‴Fe(CO)_2_}_2_(µ,η^1:1^-P_4_)] (**1**) with Ph_2_PCl and Tl[PF_6_], two sets of signals are visible for two main products—a well-resolved ADEMX spin system [31] corresponding to the *cyclo*-P_5_ unit of the novel compound [{Cp‴Fe}(µ,η^4:1^-P_5_Ph_2_){Cp‴Fe(CO)_2_}][PF_6_] (**4**, Scheme 2) and a set of broad signals with one resolved multiplet. This set of signals cannot be definitely attributed to a specific compound, but very probably contains a phosphorus core similar to that of **2a**. After removal of the solvent from the reaction mixture and extraction of the residue with a mixture of toluene and diethylether, some tiny needles of **4** could be obtained, very weakly scattering in the routine single crystal X-ray diffraction experiment but suitable for the diffraction study using high-flux synchrotron radiation (see Materials and Methods, Figure 1b). The remaining red oil was dissolved in a mixture of o-DFB and pentane. By storing the saturated solution at −30 °C, a few red crystals of [{Cp‴Fe(CO)_2_}_2_(µ,η^1:1^-P_5_Ph_2_)][PF_6_] (**3a**), suitable for X-ray diffraction analysis, could be obtained (Figure 1a). All attempts to isolate **2a** failed, due to its high instability and presumably to its rearrangement to **3a**. The presumed rearrangement of **2a** to **3a** is also supported by DFT calculations (vide infra).

**Figure 2:**
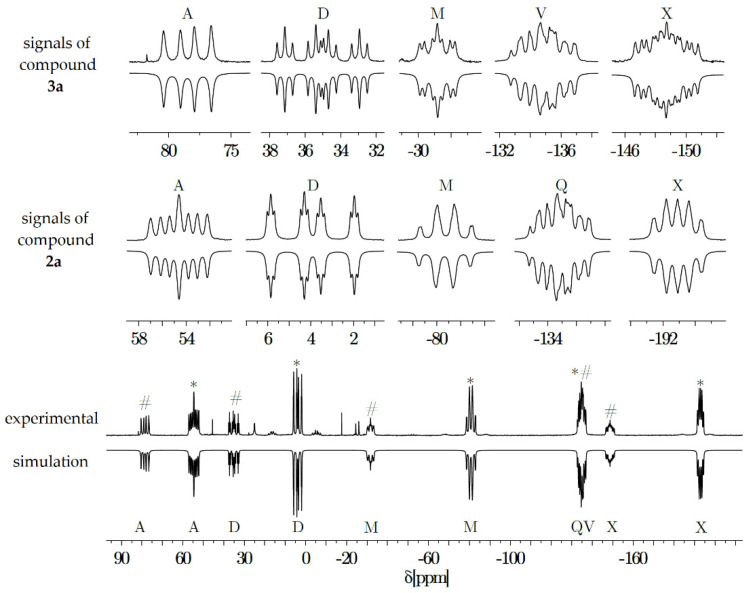
Experimental and simulated ^31^P{^1^H} NMR spectrum at 193 K of compounds **2a** and **3a** (∗ = **2a**, # = **3a**). The multiplet Q/V is the result of two overlapping multiplets from the two different spin systems.

**Figure 3:**
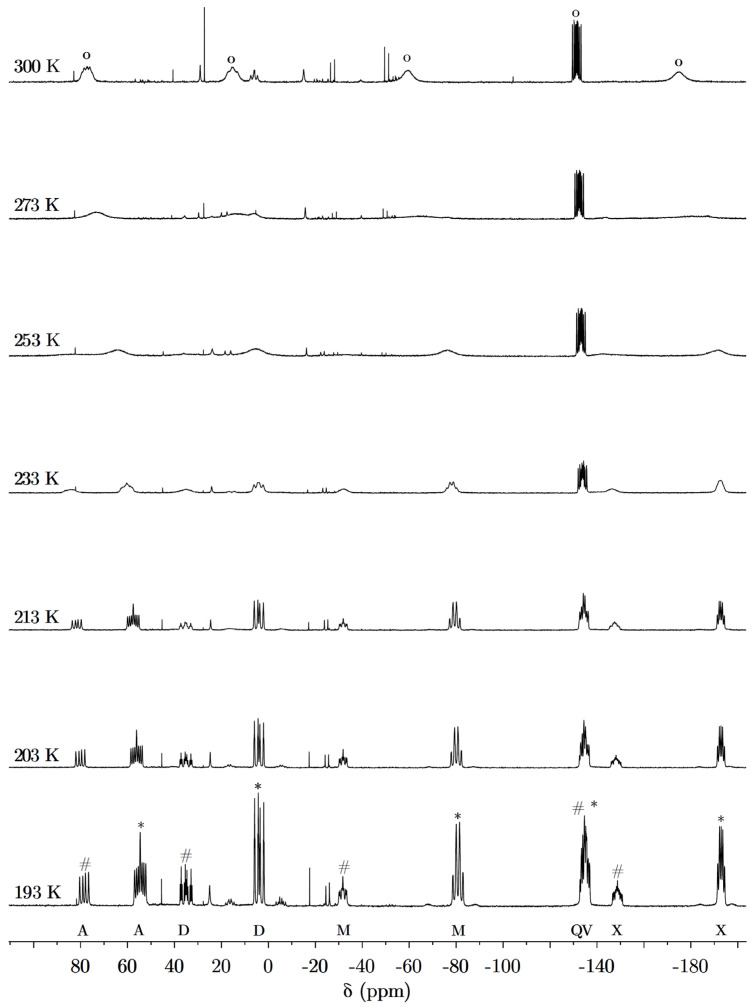
^31^P{^1^H} VT NMR spectra of the reaction solution of **1** with [Ph_2_P][OTf] in CD_2_Cl_2_ (∗ = **2a**, # = **3**, o = broad signals at r.t.).

In order to identify the intermediates which are formed in the reaction of **1** with [R_2_P]^+^ and to further investigate the nature of the dynamic behaviour in solution, ^31^P{^1^H} NMR spectroscopic studies were performed at low temperatures. For this purpose, corresponding reaction samples were prepared in dichloromethane at 193 K and kept at this temperature, without allowing them to warm up. The reaction solutions of **1** with [Ph_2_P][OTf] showed two independent spin systems, ADMVX and ADMQX, with one multiplet of each spin system overlying. The connectivity of the phosphorus cores of the two spin systems could be determined by ^31^P-^31^P COSY NMR (homonuclear correlation spectroscopy) experiments and iterative simulations (Figure 2 and Supplementary Materials). The ADMVX spin system can be attributed to **3a**, while the ADMQX spin system can very likely be attributed to **2a**. The ratio of **2a**:**3a** at 193 K was determined to be 5:2. Upon warming the sample, the signals of both spin systems broaden and collapse into each other at 253 K to give only one set of broad signals (four broad ones and one relatively sharp one, Figure 3). A further increase of the temperature only leads to a further broadening of the signals. Finally, at 300 K, four broad signals and one well-resolved one are detected in the ^31^P{^1^H} NMR spectrum. These findings show that **2a** and **3a** are formed in the first step of the reaction of **1** with [Ph_2_P]^+^, at temperatures above 253 K, however, they are in an equilibrium. The fact that one of the signals remains unaffected by the broadening within the whole temperature range, which we tentatively attribute to the Ph_2_P unit in **2a**, indicates that, at room temperature, **2a** might be the major component and that the Ph_2_P unit is interchanged between the two wingtip phosphorus atoms in a fast process on the NMR time scale. A similar behaviour was observed for [(Cp^Ar^Ni)_2_(η^3:3^-P_5_R_2_)][GaCl_4_] (Cp^Ar^ = C_5_(C_6_H_4_-4-Et)_5_, R = iPr and 2,4,6-Me_3_C_6_H_2_) [32] as well as for [{Cp‴Fe(CO)_2_}_2_(µ,η^1:1^-P_4_H)]^+^—the protonated complex of **1** [12]. According to DFT calculations in **2a**, the Ph_2_P unit binds only to one wing-tip phosphorus atom (vide infra). Increasing the temperature above 300 K leads to a rapid decomposition in dichloromethane. Unfortunately, o-DFB is not suitable for performing the experiments at 193 K due to its relatively high point of solidification (239 K).

By the reaction of [Me_2_P][OTf], generated in situ from Me_2_PCl and Tl[OTf], with **1** at 193 K in dichloromethane, the two products **2b** and **3b** could be identified in the ^31^P {^1^H} NMR spectrum at 193 K. Compound **2b** reveals an ADMQX and **3b** an ABMWX spin system. The phosphorus cores of both these compounds could be determined by iterative simulation as well as by ^31^P-^31^P COSY NMR experiments, showing the same connectivity as **2a** and **3a**. By warming the NMR sample to room temperature for the time of one measurement and subsequent cooling to 193 K, besides signs of decomposition, the relative ratio of the two products in solution changes in favour of the insertion product **3b** (**3b**:**2b** = 4:1 before r.t., 10:1 after). This suggests that either **2b** is less stable than **3b** and decomposes faster or that both compounds exist in a dynamic equilibrium at room temperature and the formation of **3b** is favoured as being the thermodynamically more stable product. For the structurally similar products **2a** and **3a**, we assume a similar behaviour (vide supra; cf. Supplementary Materials).

### 2.2. Reactions of **1** with [Ph_2_As]^+^[OTf]^−^

**Figure 4:**
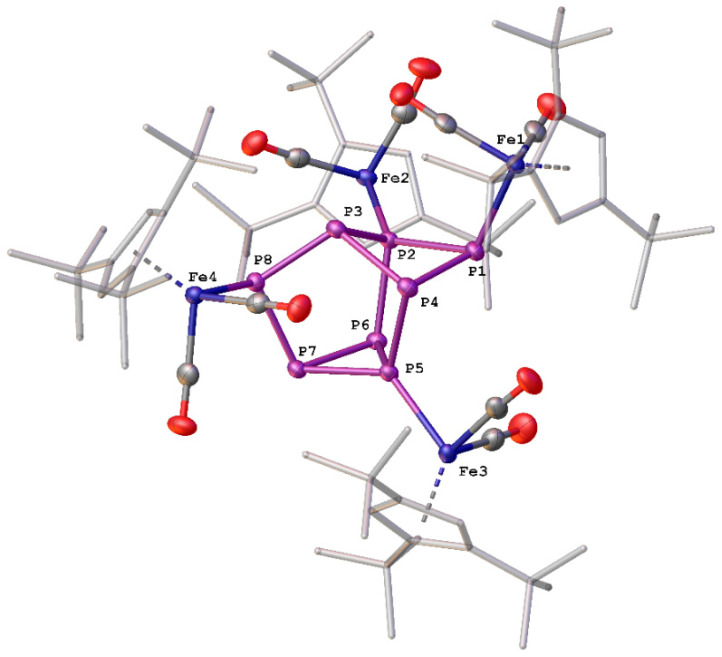
Cationic part of the molecular structure of **5** in the solid state. Hydrogen atoms, solvent molecules and counter ions are omitted for more clarity. Selected bond lengths (Å)—Fe4-P8 2.2756(5), Fe3-P5 2.2468(5), Fe2-P2 2.2587(5), Fe1-P1 2.3040(5), P5-P6 2.2115(7), P5-P7 2.1916(7), P5-P4 2.2134(7), P2-P3 2.1890(7), P2-P6 2.2713(6), P2-P1 2.2758(7), P3-P8 2.1612(7), P3-P4 2.2062(7), P6-P7 2.2071(6), P8-P7 2.1925(7), P4-P1 2.2402(7).

If **1** is reacted with [Ph_2_As][OTf] at 193 K, generated in situ from Ph_2_AsCl and Tl[OTf] in dichloromethane, two compounds showing ABX_2_ spin systems at room temperature and an ABWX for one of the compounds at 193 K are observed in the ^31^P{^1^H} NMR spectrum of the reaction solution. Iterative simulation enabled the determination of the connectivity of the phosphorus cores. However, the structural motifs of these compounds could not be undoubtedly determined, since the connectivity of the Ph_2_As fragment could not be ascertained from the NMR data. Due to their instability as well as their high solubility, these compounds could not be isolated despite several attempts. The reaction solutions in dichloromethane are only stable for a few hours at room temperature. If the reaction is performed at 193 K, the dichloromethane removed at −60 °C and the resulting dark red oil is redissolved in a mixture of o-DFB and pentane (1:1), single crystals of the complex [{Cp‴Fe(CO)_2_}_4_(µ_4_,η^1:1:1:1^-P_8_)][OTf]_2_ (**5**) can be obtained (Scheme 2 and Figure 4). Compound **5** contains a tetracyclo[3.3.0.0^2,7^.0^3,6^]-octaphosphaoctane unit which is rarely observed and represents an unexpected isomer for a P_8_ core. The only other compound reported so far containing a similar P_8_ core is [(DDP)GaBr_2_P_8_] (DDP = (2,6-diisopropylphenyl)(4-((2,6-diisopropylphenyl)imino)pent-2-en-2-yl)amide) [33]. The most common P_8_ motif is the realgar-type motif which can also be viewed as a subunit of Hittorf’s phosphorus and considered as an important building block of hierarchical structures of elemental phosphorus [34,35,36,37,38,39,40,41].

## 3. Discussion

The structural motif in the crystal structure of **3a** is identical with the postulated bicyclo[2.1.0]pentaphosphapentane framework from the low temperature NMR experiments. This structural motif is the result of the insertion of [Ph_2_P]^+^ into one of the edge-P-P bonds in **1**. The P-P bond lengths in **3a** lie in the expected range of P-P single bonds (average P-P distance in **3a**: 2.215 Å) and compare well with the P-P bond lengths in **1** [7]. The three-membered ring built up by P1, P2 and P5 (Figure 1a) and the P2P3P4P5 four-membered ring are slightly distorted. The dihedral angle between the two rings is 99° and therefore **1** is considerably more folded than **3a** (fold angle 80°). The endo-[Cp‴(CO)_2_Fe] fragment of Fe2 as well as the fragment of Fe1, remaining in an exo-position as in **1**, show a slightly shorter Fe-P bond distance (Fe1-P1 = 2.3162(12) Å and Fe2-P3 = 2.3004(15) Å) compared with the Fe-P distances in **1** (Fe1-P1 is 2.355(3) Å and Fe2-P3 is 2.346(2) Å). To the best of our knowledge, the only other compound containing a bicyclo[2.1.0]pentaphosphapentane core crystallographically characterized is the supersilylphosphine(tBu_3_Si)_3_P_5_ [42].

The formation of the *cyclo*-P_5_ unit in **4** (Figure 1b) is the result of the twofold decarbonylation of one iron center (probably Fe2) in **3a** (Figure 1a) with a consequent opening of the P2-P6 bond. As an η^4^-coordinating *cyclo*-P_5_ ligand with one phosphorus atom bent out of the plane, the *cyclo*-P_5_ unit in **4** can be compared to the known P_5_ ligand in [K(dme)K(dibenzo-[18]crown-6)][Cp*Fe(η^4^-P_5_)] [43], the functionalized P_5_-ligands in [Cp*Fe(η^4^-P_5_R)] (R = NMe_2_, PH_2_, CH_2_SiMe_3_) [44] or the cationic complex [Cp^Ar^Co(η^4^-P_5_R_2_)][GaCl_4_] (Ar = C_5_(C_6_H_4_-4-Et)_5_, R = iPr and Cy) [32], but differs in bond lengths and angles due to its cationic character and its coordination of a second iron fragment. This is also the reason why **4** shows a complex ADEMX spin system for an asymmetric P_5_ moiety of magnetically inequivalent phosphorus atoms instead of an AMM’XX’ spin system as expected for the aforementioned compounds. In **4**, the bond distances of the Ph_2_P fragment P1-P2 (2.1761(9) Å) and P1-P5 (2.1590(9) Å) to the rest of the P_5_ moiety are noticeably different. The other bond distances are significantly shorter, with P2-P3 being the shortest one (2.1293(9) Å). Apart from the lack of symmetry, the bond lengths are also in agreement with the known *cyclo*-P_5_ moieties mentioned above.

**Scheme 3:**
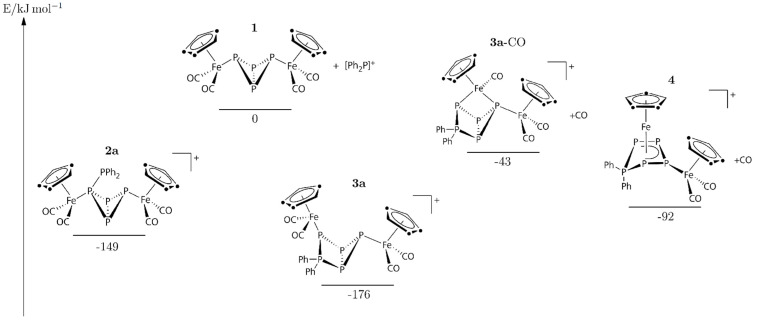
Relative energies of starting material **1** and [PPh_2_]^+^, **3a** and its decarbonylation to **4** on the BP86/def2-SVP level.

According to the variable temperature NMR experiments, the reaction mixtures of **1** with phosphenium ions turned out to be stable only up to −60 °C in dichloromethane. By changing the solvent and the anion in order to find a reaction set up stable enough to obtain the single crystals of **3a** and **4**, respectively, the cationic parts of **2a** and **3a** were found to be more stable in o-DFB, using stronger coordinating counter ions such as [OTf]^−^. Using weakly coordinating anions such as [TEF]^−^ ([TEF]^−^ = [Al(OC(CF_3_)_3_)_4_]^−^) [45] leads to less stable reaction mixtures or to the formation of larger quantities of **4** in dichloromethane. Using o-DFB and the [PF_6_]^−^ anion, the products were stable enough for crystallization, and small amounts of single crystals of **3a** and **4** suitable for X-ray diffraction studies could be obtained. This leads to the presumption that the cationic parts of **2a** and **3a** are not stable enough without being stabilized by a coordinating anion so that at least **3a** undergoes two consecutive decarbonylations to form **4** (Scheme 3). The decarbonylation of [{Cp″Fe(CO)_2_}_2_(µ,η^1:1^-P_4_)] leading to [{Cp″Fe(CO)_2_}(µ,η^1:2^-P_4_){Cp″Fe(CO)}] was reported [46]. To clarify if **4** was formed via the decarbonylation of **3a**, DFT calculations at the BP86/def2-SVP level were performed. The results show that the attack of the phosphenium ion is exergonic, while the first decarbonylation of **3** is noticeably endergonic. The fact that the second decarbonylation is exergonic would explain why the product from the first decarbonylation of **3a** could not be detected, except in the ESI-MS spectra of freshly prepared reaction solutions. At room temperature, the reaction energy released from the formation of **3a** was large enough to trigger the first decarbonylation to some degree, which would explain the formation of **4** at room temperature as well as the absence of **4** when reaction solutions are prepared at low temperatures and with stabilizing anions. It is uncertain so far whether **2a** undergoes a twofold decarbonylation at room temperature as well. Since ^31^P NMR experiments showed that **2b** and **3b** are not equally stable upon varying the temperature and because **2a** and **3a** exhibited a similar behaviour, DFT calculations were performed on **2a**. Their results also show that the cationic part in **3a** is 27 kJ/mol lower in energy than **2a**.

The variation of chlorophosphine to dimethyl-, dicyclohexyl-, ditertbutyl- or bis-diethylamino-chlorophosphine or bis-benzo-chlorophosphole only leads to satisfactory results in the case of dimethyl-chlorophosphine giving similar structural motifs in the low-temperature ^31^P NMR experiments as the phenyl-substituted derivatives **2a** and **3a**. Compounds **2b** and **3b** show an even higher sensitivity than the phenyl-substituted compounds. The products formed by the coordination of [Ph_2_As]^+^ to **1** are even less stable and convert to compound **5** and Ph_4_As_2_. Compound **5** can formally be described as the dimer of the radical cation of **1**. As already mentioned, the P_8_ unit in **5** is a very rare example of a tetracyclo[3.3.0.0^2,7^.0^3,6^]octaphosphaoctane core, which had only been observed in [(DDP)GaBr_2_P_8_] [33]. The P-P bond lengths in the solid-state structure of **5** are very similar and vary from 2.1612(7) Å (P3-P8) to 2.2758(7) Å (P2-P1). The ^31^P NMR spectrum of **5** shows six broadened and two sharp multiplets of an AEGHMQTX spin system of eight chemically inequivalent phosphorus atoms. In order to assign the signals properly to the corresponding phosphorus atoms in the solid-state structure, we conducted variable temperature NMR experiments (193 to 300 K, Supplementary Materials). These experiments did not result in freezing any dynamic process in solution to the extent that the spin system is resolved well enough on the NMR time scale for iterative simulation. However, the couplings were resolved well enough for ^31^P-^31^P COSY NMR measurements from which the connectivity of the P_8_ moiety in **5** could be determined. The general principle of formally oxidizing compounds by attacking the diphenylarsenium cations and consequently forming tetraphenyldiarsine as a thermodynamic driving force should be applicable to electron-rich starting materials other than **1** and prove to be a good starting point for future studies.

## 4. Materials and Methods

### 4.1. General Techniques and Materials

All manipulations were performed using standard Schlenk techniques on a dual manifold Schlenk line or a glove box under an atmosphere of dry argon. Traces of oxygen in the inert gas were removed by passing it over a BASF R 3-11 (CuO/MgSiO_3_) catalyst. Residual moisture in the inert gas was removed, by passing it through concentrated H_2_SO_4_, orange gel and sicapent on dried pumice stone. All glassware was vigorously heated in dynamic vacuum before use and any Teflon tubing was stored in an oven at 443 K. Dichloromethane, toluene, hexane and pentane were purified and dried by the solvent purification system SPS-800 from MBRAUN (Garching, Germany). Deuterated solvents and o-DFB were dried and distilled over CaH_2_. All solvents were stored over a molecular sieve, which was dried in a dynamic vacuum at 623 K. Ph_2_PCl, Me_2_PCl, TlPF_6_ and TlOTf were obtained from Sigma Aldrich (St. Louis, MO, USA) and used without further purification, except for Ph_2_PCl, which was distilled prior to use. Me_2_PCl and its standard solutions were stored and handled at temperatures below 277 K at all times. [{Cp‴Fe(CO)_2_}_2_(µ,η^1:1^-P_4_)] [8] and Ph_2_AsCl [47] were synthesized according to literature procedures. NMR spectra were measured with a Bruker Avance 400 (Billerica, MA, USA) (^1^H: 400.13 MHz; ^13^C: 100.61 MHz; ^31^P: 161.97 MHz) using special NMR tubes with a screw cap or a J. Young valve. Any working steps preparing samples for measurement at 193 K were performed at this temperature and the samples were stored at 193 K until measured. The spin systems of the ^31^P NMR spectra were simulated with the DAISY package in TopSpin 3.2 from Bruker (Billerica, MA, USA) using the data from 2D NMR spectra to assign the multiplets to the corresponding compounds and obtain first insights into the connectivity of the phosphorus framework. All coupling constants are given in Hz. The diffraction from a tiny needle-shaped single crystal of **4**, of 4 × 10 × 50 μm^3^ in size, was collected at 80 K on the P11 beamline at PETRAIII synchrotron at DESY (Hamburg, Germany) [48], using the high-flux X-ray beam focused and collimated down to 50 μm in ø to reduce the background and to significantly improve the intensity-to-noise ratio.

### 4.2. Reaction of **1** with in Situ Generated [Ph_2_P][PF_6_] at Room Temperature

To a solution of [{Cp‴Fe(CO)_2_}_2_(µ,η^1:1^-P_4_)] (**1**) (82 mg, 0.1 mmol) and TlPF_6_ (36 mg, 0.103 mmol) in 5 mL o-DFB, a standard solution of Ph_2_PCl in toluene (c = 0.2 mmol mL^−1^) was added at room temperature. While stirring for 12 h, a colourless precipitate of TlCl was formed from which a dark red solution was decanted. The solvent was carefully removed and the resulting red oil extracted with a mixture of diethyl ether and toluene (10:1, Et_2_O:toluene) (three times with 5 mL). The pale red diethyl ether/toluene solution was stored at 277 K for 2 months. Some extremely fine needles of **4** suitable for diffraction with synchrotron radiation were retrieved from the flask. The residual oil was redissolved in o-DFB at room temperature and, upon addition of pentane at 243 K, the formation of a dark red oil could be observed. After storage at room temperature until the oil redissolved, the flask was stored at 243 K for crystallization. After one month, a few crystals of **3a** suitable for X-ray diffraction studies could be obtained. The amounts of crystalline **4** and **3a** were too small to estimate the yield of the isolated products. ^31^P{^1^H} NMR spectra of the reaction solution show full conversion of **1**. ^1^H NMR of reac. mix.—(CD_2_Cl_2_, 300 K): δ(ppm) = 1.02 (m, 54H, C_5_H_2_(C(CH_3_)_3_)_3_), 4.84 (m, 4H, C_5_H_2_(C(CH_3_)_3_)_3_), 7.40 (m, 10H, P(C_6_H_5_)_2_); IR of reac. mix.—(CH_2_Cl_2_, 300 K): ν(cm^−1^) = 1975 (s), 2016 (s); ESI-MS—*m*/*z* = 1001 ([M]^+^), 971 ([M]^+^-CO), 943 ([M]^+^-2CO); ^31^P{^1^H} NMR of **4** (CD_2_Cl_2_, 300 K)—δ(ppm) = 210.8 (m, P_A_, ^1^J_PAPM_ = 365, ^1^J_PAPX_ = 365, ^2^J_PAPD_ = 26), 150.6 (m, P_D_, ^1^J_PDPE_ = 415, ^1^J_PDPX_ = 370, ^2^J_PDPA_ = 26, ^2^J_PDPM_ = 14), 125.4 (m, Ph_2_P_E_, ^1^J_PEPD_ = 415, ^1^J_PEPM_ = 516, ^2^J_PEPX_ = 11), 53.9 (m, P_M_, ^1^J_PMPA_ = 365, ^1^J_PMPE_ = 516, ^2^J_PMPD_ = 14, ^2^J_PMPX_ = 16), 53.9 (m, P_X_, ^1^J_PXPA_ = 390, ^1^J_PXPD_ = 370, ^2^J_PXPE_ = 11, ^2^J_PXPM_ = 16).

### 4.3. Low Temperature NMR Experiments

To a solution of [{Cp‴Fe(CO)_2_}_2_(µ,η^1:1^-P_4_)] (**1**) (100 mg, 0.122 mmol) and TlOTf (65 mg, 0.183 mmol) in 5 mL dichloromethane, a standard solution of a chlorophosphine R_2_PCl (R = Ph, Me, 0.122 M) in toluene was added at 193 K. While stirring for 5 h, a colourless precipitate of TlCl was formed from which the dark red solution was quickly but meticulously decanted at 193 K into a cooled Schlenk flask after sedimentation. The solvent was carefully removed at 193 K, the dark red oil redissolved in precooled CD_2_Cl_2_ and was transferred into a precooled NMR tube with a J. Young valve, maintaining 193 K at all times. For mass spectrometry, the time between removing the sample (before removing the solvent) from the cooled Schlenk and the injection was minimized to ca. 2 min. ^31^P{^1^H} NMR spectroscopic data show full conversion of **1**. ^1^H NMR of reac. mix. for R = Ph—(CD_2_Cl_2_, 300 K): δ(ppm) = 1.39–1.57 (m, 54H, C_5_H_2_(C(CH_3_)_3_)_3_), 5.18 (m, 4H, C_5_H_2_(C(CH_3_)_3_)_3_), 7.68–8.15 (m, 10H, P(C_6_H_5_)_2_); ESI-MS—*m*/*z* = 1001 ([M]^+^), 971 ([M]^+^-CO), 943 ([M]^+^-2CO); ^31^P{^1^H} NMR of **2a** (CD_2_Cl_2_, 193 K)—δ(ppm) = 54.5 (m, P_A_, ^1^J_PAPD_ = 379, ^1^J_PAPM_ = 256, ^1^J_PAPX_ = 134, ^2^J_PAPQ_ = 19), 3.9 (m, P_D_, ^1^J_PDPA_ = 379, ^1^J_PDPQ_ = 254, ^2^J_PDPM_ = 29, ^2^J_PDPA_ = 24), −80.7 (m, P_M_, ^1^J_PMPA_ = 256, ^1^J_PMPQ_ = 211, ^1^J_PMPX_ = 228, ^2^J_PMPD_ = 29), −135.0 (m, P_Q_, ^1^J_PQPD_ = 254, ^1^J_PQPM_ = 211, ^1^J_PQPX_ = 122, ^2^J_PQPA_ = 19), −192.8 (m, P_X_, ^1^J_PXPA_ = 134, ^1^J_PXPM_ = 228, ^1^J_PXPQ_ = 122, ^2^J_PXPD_ = 24); ^31^P{^1^H} NMR of **3a** (CD_2_Cl_2_, 193 K)—δ(ppm) = 78.4 (m, P_A_, ^1^J_PAPD_ = 390, ^1^J_PAPX_ = 218), 35.0 (m, P_D_, ^1^J_PDPA_ = 390, ^1^J_PDPV_ = 283, ^2^J_PDPM_ = 76, ^1^J_PDPX_ = 68), −31.7 (m, P_M_, ^1^J_PMPV_ = 190, ^1^J_PMPX_ = 260, ^2^J_PMPD_ = 76), −134.5 (m, P_V_, ^1^J_PVPD_ = 283, ^1^J_PVPM_ = 190, ^1^J_PVPX_ = 119), −148.7 (m, P_X_, ^1^J_PXPA_ = 218, ^1^J_PXPM_ = 260, ^1^J_PXPV_ = 119, ^2^J_PXPD_ = 68); ^1^H NMR of reac. mix. for R = Me—(CD_2_Cl_2_, 193 K): δ(ppm) = 1.42–1.57 (m, 54H, CpH_2_(C(CH_3_)_3_)_3_), 2.00 (m, 6H, P(CH_3_)_2_), 5.15–5.31 (m, 4H, CpH_2_(C(CH_3_)_3_)_3_); IR of reac. mix. for R = Me—(CH_2_Cl_2_, 300 K): ν(cm^−1^) = 1966 (s), 2015 (s), 2046 (s); ESI-MS—*m*/*z* = 875 ([{Cp’’’Fe(CO)_2_}_2_(µ,η1:1-P4)]+[Me_2_P]^+^), 847 ([M]^+^-CO), 819 ([M]^+^-2CO); ^31^P{^1^H} NMR of **2b** (CD_2_Cl_2_, 193 K)—δ(ppm) = 45.6 (m, P_A_, ^1^J_PAPB_ = 352, ^1^J_PAPW_ = 223, ^1^J_PAPB_ = 352), 37.4 (m, P_B_, ^1^J_PBPA_ = 352, ^1^J_PBPM_ = 265, ^1^J_PBPX_ = 106, ^2^J_PBPW_ = 60), −48.6 (m, P_M_, ^1^J_PMPB_ = 265, ^1^J_PMPX_ = 192, ^2^J_PMPW_ = 99), −146.0 (m, P_W_, ^1^J_PWPA_ = 223, ^1^J_PWPX_ = 261, ^2^J_PWPB_ = 60, ^2^J_PWPM_ = 99), −150.7 (m, P_X_, ^1^J_PXPB_ = 106, ^1^J_PXPM_ = 192, ^1^J_PXPW_ = 261); ^31^P{^1^H} NMR of **3b** (CD_2_Cl_2_, 193 K)—δ(ppm) = 39.6 (m, P_A_, ^1^J_PAPD_ = 353, ^1^J_PAPM_ = 243, ^1^J_PAPX_ = 130, ^2^J_PAPQ_ = 25), 2.1 (m, P_D_, ^1^J_PDPA_ = 353, ^1^J_PDPQ_ = 244, ^2^J_PDPM_ = 32, ^1^J_PDPX_ = 26), −82.6 (m, P_M_, ^1^J_PMPA_ = 243, ^1^J_PMPQ_ = 206, ^1^J_PMPX_ = 237, ^2^J_PMPD_ = 32), −140.2 (m, P_Q_, ^1^J_PQPD_ = 244, ^1^J_PQPM_ = 206, ^1^J_PQPX_ = 114, ^2^J_PQPA_ = 25), −182.3 (m, P_X_, ^1^J_PXPA_ = 130, ^1^J_PXPM_ = 237, ^1^J_PXPQ_ = 114, ^2^J_PXPD_ = 26).

### 4.4. Reaction of **1** with in Situ Generated [Ph_2_As][OTf] at 193 K

To a mixture of [{Cp‴Fe(CO)_2_}_2_(µ,η^1:1^-P_4_)] (**1**) (100 mg, 0.122 mmol), TlOTf (65 mg, 0.183 mmol) and Ph_2_AsCl (32 mg, 0.122 mmol), 10 mL of precooled dichloromethane was added at 193 K under stirring. After one hour of stirring and storing the mixture for two days at 193 K to let the colourless solid settle, the vibrant red solution is decanted via short Teflon tubing into a precooled Schlenk flask. After removing the solvent at 193 K, a viscous, dark red oil is formed. ^31^P{^1^H} NMR spectra of the reaction solution showed full conversion of **1**. Redissolving in o-DFB and adding the same volume of pentane to the dark red solution yielded 54 mg (0.027 mmol, yield 44% of theory) dark red crystals of **5** after storage at room temperature for 24 h. ^1^H NMR of reac. Mix—(CD_2_Cl_2_, 193 K): δ(ppm) = 1.27 (m, 54H, C_5_H_2_(C(CH_3_)_3_)_3_), 4.89 (m, 2H, C_5_H_2_(C(CH_3_)_3_)_3_), 4.99 (m, 2H, C_5_H_2_(C(CH_3_)_3_)_3_), 7.54 (m, 10H, As(C_6_H_5_)_2_); ^31^P{^1^H} NMR of coordination mode 1 of [Ph_2_As][OTf] on **1**—(CD_2_Cl_2_, 193 K): δ(ppm) = 57.7 (m, P_A_, ^1^J_PAPW_ = 259, ^1^J_PAPX_ = 244, ^2^J_PAPB_ = 24), 43.8 (m, P_B_, ^1^J_PBPW_ = 214, ^1^J_PBPX_ = 218, ^2^J_PBPA_ = 24), −237.0 (m, P_W_, ^1^J_PWPA_ = 259, ^1^J_PWPB_ = 214, ^1^J_PWPX_ = 109), −250.6 (m, P_X_, ^1^J_PXPA_ = 244, ^1^J_PXPB_ = 218, ^1^J_PXPW_ = 109); ^31^P{^1^H} NMR of coordination mode 2 of [Ph_2_As][OTf] on **1** (CD_2_Cl_2_, 193 K)—δ(ppm) = −10.9 (m, P_A_, ^1^J_PAPB_ = 125, ^1^J_PAPX_ = 203), −12.1 (m, P_B_, ^1^J_PBPA_ = 125, ^1^J_PBPX_ = 256), −274.9 (m, 2P_X_, ^1^J_PXPA_ = 203, ^1^J_PXPB_ = 256); Analytical data for isolated crystals of **5**: EA—Expected for (C_78_H_116_F_6_Fe_4_O_14_P_8_S_2_ (**5**) + 0.6 C_6_H_4_F_2_): 49.11 C, 5.98% H, 3.21% S. Found: 49.64% C, 5.70% H, 3.72% S; IR: ν(cm^−1^) = 1964 (s), 1989 (s), 2029 (s), 2032 (s); ESI-MS—*m*/*z* = 826 ([M]^2+^); ^1^H NMR (CD_2_Cl_2_, 300 K)—δ(ppm) = 1.21–1.54 (m, 108H, CpH_2_(C(CH_3_)_3_)_3_), 4.57–5.65 (m, 8H, CpH_2_(C(CH_3_)_3_)_3_); ^31^P NMR (CD_2_Cl_2_, 300 K)—δ(ppm) = 244.3 (mbr, P_A_), 183.3 (mbr, P_E_), 136.3 (m, P_G_), 128.4 (mbr, P_H_), 69.5 (mbr, P_M_), 40.3 (mbr, P_Q_), 2.5 (mbr, P_T_), −165.4 (m, P_X_).

## 5. Conclusions

It could be shown that the insertion of phosphenium ions into the bicylo-[1.1.0]-tetraphosphabutane unit of [{Cp‴Fe(CO)_2_}_2_(µ,η^1:1^-P_4_)] (**1**) is possible. Due to the sensitivity of the reaction solutions, extensive low-temperature NMR studies involving ^31^P-^31^P COSY NMR and iterative simulation of the complex spin systems were performed to ascertain the connectivity of the phosphorus cores of the compounds formed at 193 K. These resulted in the characterization of a 2-diphenylphosphabicyclo[1.1.0]tetraphosphabutane unit in **2a** as well as a rarely observed bicyclo[2.1.0]pentaphosphapentane unit in [{Cp‴Fe(CO)_2_}_2_(µ,η^1:1^-P_5_(C_6_H_5_)_2_)][PF_6_] (**3a**), which could be confirmed by single crystal X-ray diffraction. Regardless of the low isolated yield of crystalline **3a** and **4**, the ^31^P NMR spectra of freshly prepared reaction solutions show full conversion of **1** to the corresponding products. Additionally, DFT computations were used to gain more insight into the decarbonylation process of **3a** to the cationic compound [{Cp‴Fe}(µ,η^4:1^-P_5_(C_6_H_5_)_2_){Cp‴(CO)_2_Fe}][PF_6_] (**4**). Furthermore, the reactivity of diphenylarsenium ions towards **1** was investigated, showing two temperature-sensitive coordination products by applying low-temperature NMR studies. By exposing solutions of these product mixtures to room temperature, the formation of Ph_4_As_2_ and the disproportionation product [{Cp‴Fe(CO)_2_}_4_(µ_4_η^1:1:1:1^-P_8_)][OTf]_2_ (**5**) with a rarely found tetracyclo[3.3.0.0^2,7^.0^3,6^]octaphosphaoctane unit could be observed.

## Data Availability

The data presented in this study are available in supplementary material.

## Supplementary Materials

The following are available online. NMR spectra with the assignment of phosphorus atoms, details of the crystal structure refinements and the DFT calculations.
