# Supplementary material for: Insertion of Phosphenium Ions into a Bicyclo[1.1.0]Tetraphosphabutane Iron Complex
Source: Molecules. 2021 Jun 26;26(13):3920. doi: 10.3390/molecules26133920 (PMC8272002; doi:10.3390/molecules26133920)
Supplement: Supplementary file 1 [file molecules-26-03920-s001.zip › molecules-1274738-supplementary.pdf]

Insertion of phosphenium ions into an iron stabilized bicyclo[1.1.0]tetraphosphabutane motif

Supplementary Information

Martin Weber [1], Gábor Balázs [1], Alexander V. Virovets [1], Eugenia Peresypkina [1] and Manfred Scheer [1,*]

[1] Institute of Inorganic Chemistry, University of Regensburg, Universitätsstrasse 31, 93040 Regensburg (Germany)

* Correspondence: manfred.scheer@ur.de

Contents

Supplementary Information 1

1 NMR data and spectra 2

1.1 NMR data from simulations 2

1.2 NMR spectra of reactions of **1** with $[Ph_2P][OTf]$ at 193 K 4

1.3 NMR spectra of reactions of **1** with $[Me_2P][OTf]$ at 193 K 9

1.4 NMR spectra of the reaction of **1** with in situ generated $[Ph_2P][PF_6]$ at room temperature 13

1.5 NMR spectra of the reaction of **1** with in situ generated $[Ph_2As][OTf]$ ... 15

2 Crystallographic details 21

2.1 Crystallographic details on **3a** 21

2.2 Crystallographic details on **4** 21

2.3 Crystallographic details on **5** 22

2.4 Crystallographic data 23

2.5 Solid state structures 24

3 Details on DFT-calculations 27

3.1 General remarks 27

3.2 Thermodynamic data from frequency calculations 27

3.3 Cartesian coordinates of optimised structures 27

Appendix 41

List of Figures 41

List of Tables 42

References 43

1 NMR data and spectra

1.1 NMR data from simulations

Table S1: $^{31}P\{^{1}H\}$ NMR data of **2a**: (CD_2Cl_2; 193 K) with assignment of the individual phosphorus atoms:

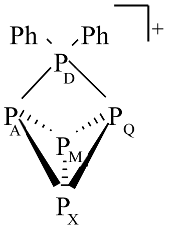

	δ (ppm)	J (Hz)			
P_A	54.5	$^1J_{P_AP_D}$	379	$^1J_{P_MP_X}$	228
P_D	3.9	$^1J_{P_AP_M}$	256	$^1J_{P_QP_X}$	122
P_M	-80.7	$^1J_{P_AP_X}$	134	$^2J_{P_AP_Q}$	19
P_Q	-135.0	$^1J_{P_DP_Q}$	254	$^2J_{P_DP_M}$	29
P_X	-192.8	$^1J_{P_MP_Q}$	211	$^2J_{P_DP_X}$	24

Table S2: $^{31}P\{^{1}H\}$ NMR data of **3a**: (CD_2Cl_2; 193 K) with assignment of the individual phosphorus atoms:

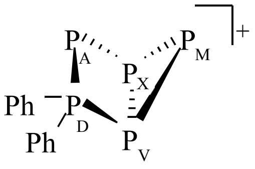

	δ (ppm)	J (Hz)			
P_A	78.4	$^1J_{P_AP_D}$	390	$^1J_{P_VP_X}$	119
P_D	35.0	$^1J_{P_AP_X}$	218	$^2J_{P_DP_M}$	76
P_M	-31.7	$^1J_{P_DP_V}$	283	$^2J_{P_DP_X}$	68
P_V	-134.5	$^1J_{P_MP_V}$	190		
P_X	-148.7	$^1J_{P_MP_X}$	260		

Table S3: $^{31}P\{^{1}H\}$ NMR data of **2b**: (CD_2Cl_2; 193 K) with assignment of the individual phosphorus atoms:

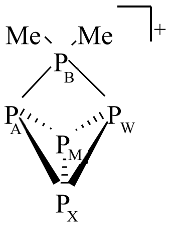

	δ (ppm)	J (Hz)			
P_A	45.6	$^1J_{P_AP_B}$	352	$^1J_{P_WP_X}$	261
P_B	37.4	$^1J_{P_AP_W}$	223	$^2J_{P_BP_W}$	60
P_M	-48.6	$^1J_{P_BP_M}$	265	$^2J_{P_MP_W}$	99
P_W	-146.0	$^1J_{P_BP_X}$	106		
P_X	-150.7	$^1J_{P_MP_X}$	192		

Table S4: ^{31}P{^{1}H} NMR data of **3b**: (CD_2Cl_2; 193 K) with assignment of the individual phosphorus atoms:

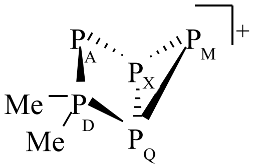

	δ (ppm)	J (Hz)			
P_A	39.6	$^{1}J_{P_AP_D}$	353	$^{1}J_{P_MP_X}$	237
P_D	2.1	$^{1}J_{P_AP_M}$	243	$^{1}J_{P_QP_X}$	114
P_M	-82.6	$^{1}J_{P_AP_X}$	130	$^{2}J_{P_AP_Q}$	25
P_Q	-140.2	$^{1}J_{P_DP_Q}$	244	$^{2}J_{P_DP_M}$	32
P_X	-182.3	$^{1}J_{P_MP_Q}$	206	$^{2}J_{P_DP_X}$	26

Table S5: ^{31}P{^{1}H} NMR data of **4**: (CD_2Cl_2; 300 K) with assignment of the individual phosphorus atoms:

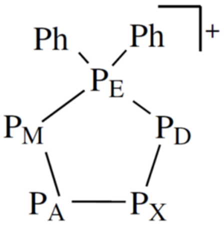

	δ (ppm)	J (Hz)			
P_A	210.8	$^{1}J_{P_AP_M}$	365	$^{2}J_{P_AP_D}$	26
P_D	150.6	$^{1}J_{P_AP_X}$	390	$^{2}J_{P_DP_M}$	14
P_E	125.4	$^{1}J_{P_DP_E}$	415	$^{2}J_{P_EP_X}$	11
P_M	53.9	$^{1}J_{P_DP_X}$	370	$^{2}J_{P_MP_X}$	16
P_X	-25.6	$^{1}J_{P_EP_M}$	516		

Table S6: ^{31}P{^{1}H} NMR data of coordination mode 1 of [Ph_2As][OTf] on **1** from the simulation (in CD_2Cl_2 at 193 K).

	δ (ppm)	J (Hz)			
P_A	57.7	$^{1}J_{P_AP_W}$	259	$^{1}J_{P_BP_X}$	218
P_B	43.8	$^{1}J_{P_AP_X}$	244	$J_{P_WP_X}$	109
P_W	-237.0	$^{1}J_{P_BP_W}$	214	$^{2}J_{P_AP_B}$	24
P_X	-250.6				

Table S7: ^{31}P{^{1}H} NMR data of coordination mode 2 of [Ph_2As][OTf] on **1** from the simulation (in CD_2Cl_2 at 193 K).

	δ (ppm)	J (Hz)	
P_A	-10.9	$^{1}J_{P_AP_B}$	125
P_B	-12.1	$^{1}J_{P_AP_{X_2}}$	203
P_{X_2}	-274.9	$^{1}J_{P_BP_{X_2}}$	256

1.2 NMR spectra of reactions of **1** with [Ph_2P][OTf] at 193 K

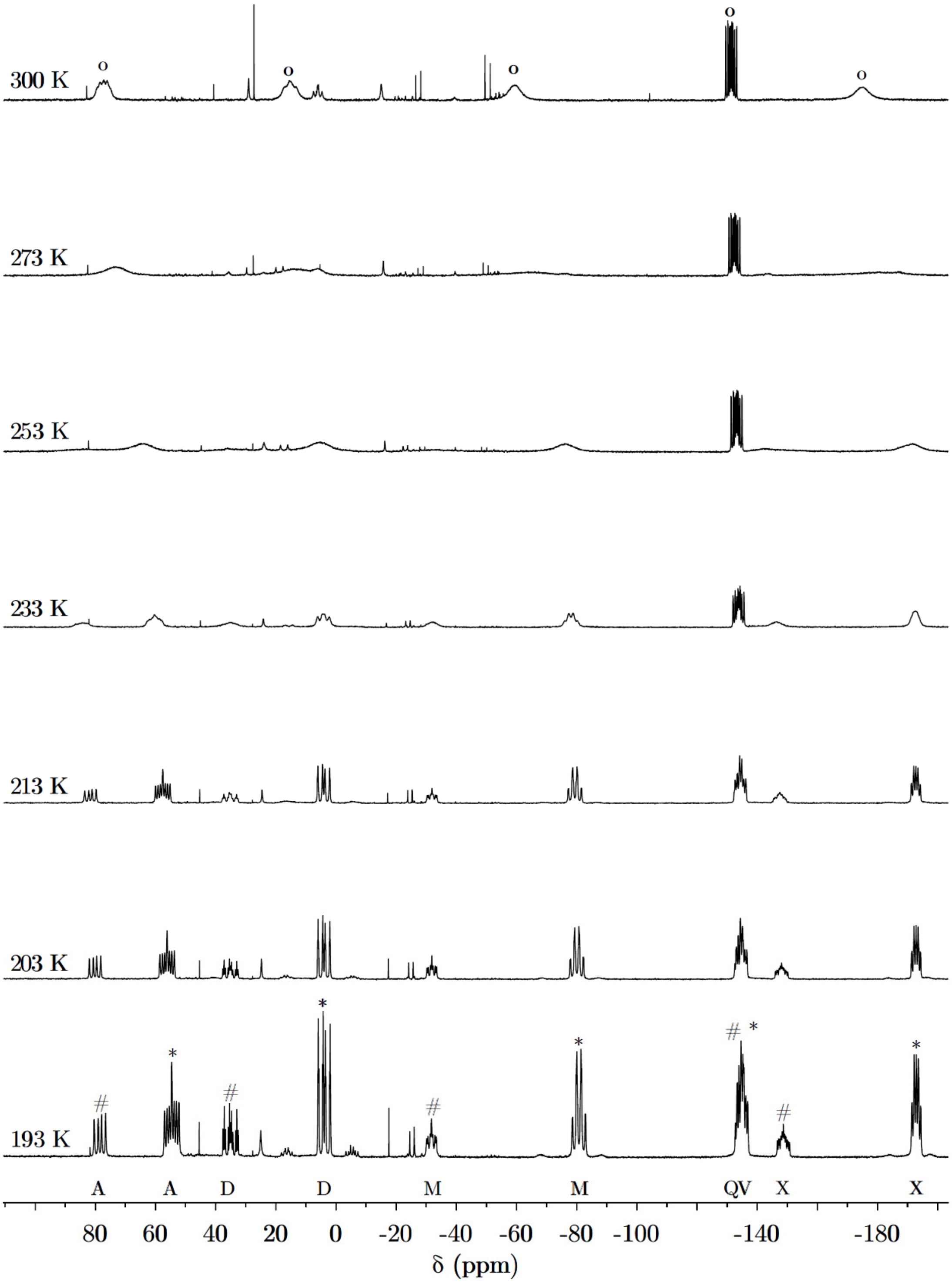

Figure S1: $^{31}P\{^1H\}$ VT NMR spectrum in CD_2Cl_2 from the reaction of **1** with [Ph_2P][OTf]. The signals of compound **2a** are marked with ∗, of compound **3a** with # and signals showing dynamic behaviour at room temperature with o.

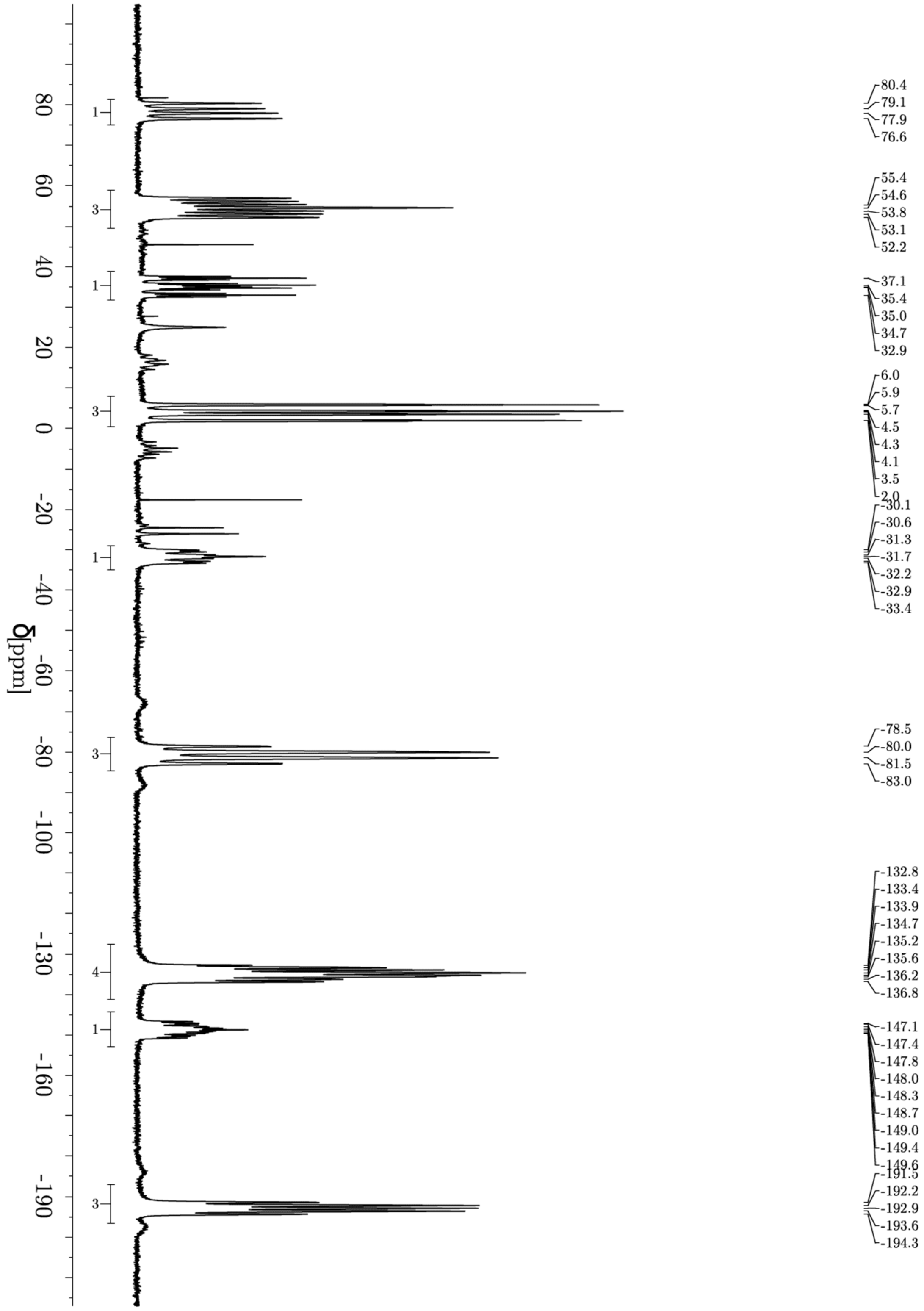

Figure S2: $^{31}P\{^{1}H\}$ NMR spectrum (excerpt) at 193 K in CD_2Cl_2 from the reaction of **1** with $[Ph_2P][OTf]$ at 193 K with chemical shifts and integrals depicted in the spectrum.

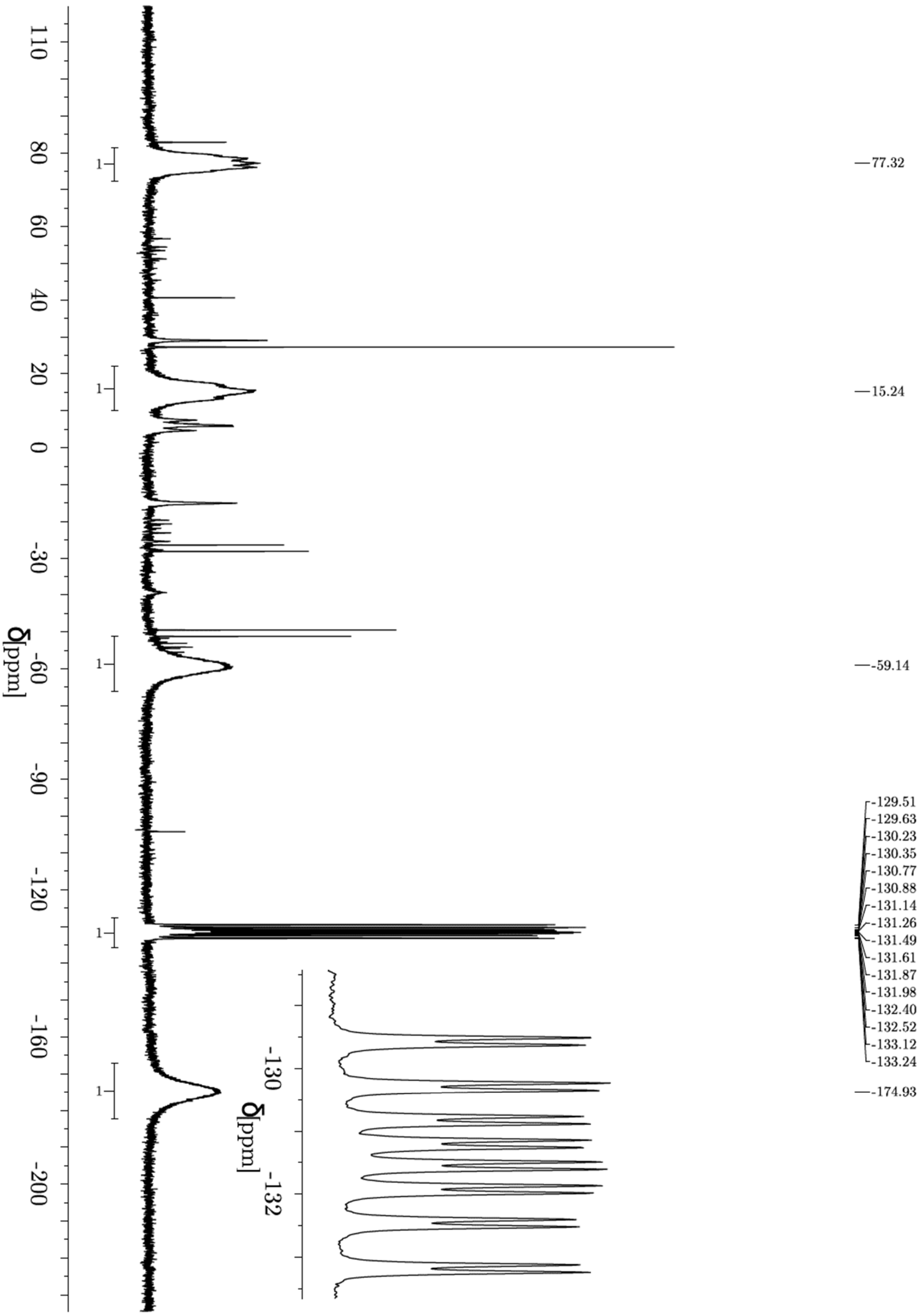

Figure S3: $^{31}P\{^{1}H\}$ NMR spectrum (excerpt) at room temperature in CD_2Cl_2 from the reaction of **1** with $[Ph_2P][OTf]$ at 193 K with chemical shifts and integrals depicted in the spectrum.

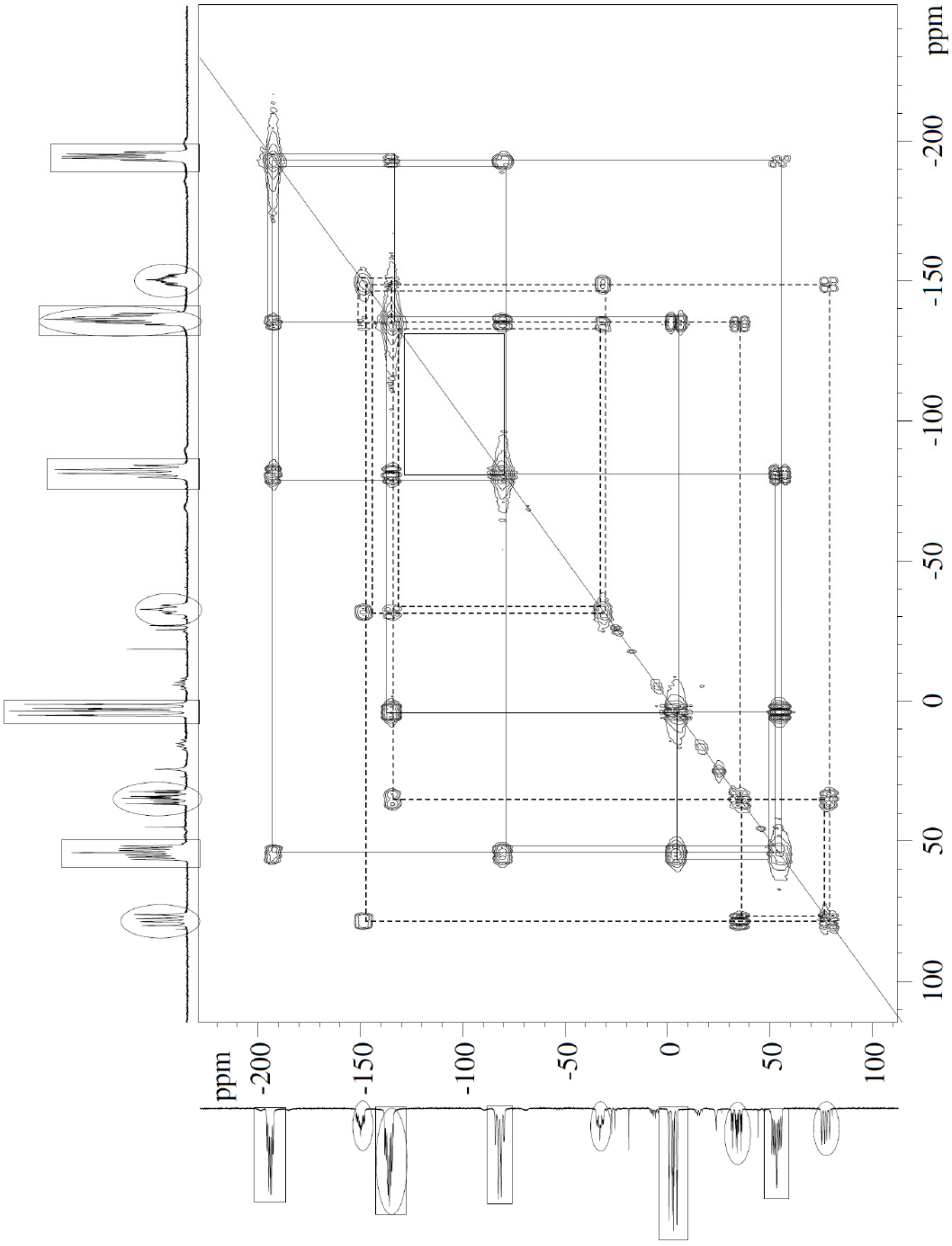

Figure S4: ^{31}P-^{31}P COSY NMR spectrum in CD_2Cl_2 at 193 K of the product mixture from the reaction of $[Ph_2P][OTf]$ with **1** at 193 K. Signals of **2a** are marked with rectangles, of **3a** with ellipses. Couplings in compound **2a** are drawn as rectangles with solid lines, couplings in **3a** with dashed lines. For easier understanding, the diagonal signals are connected via a solid line.

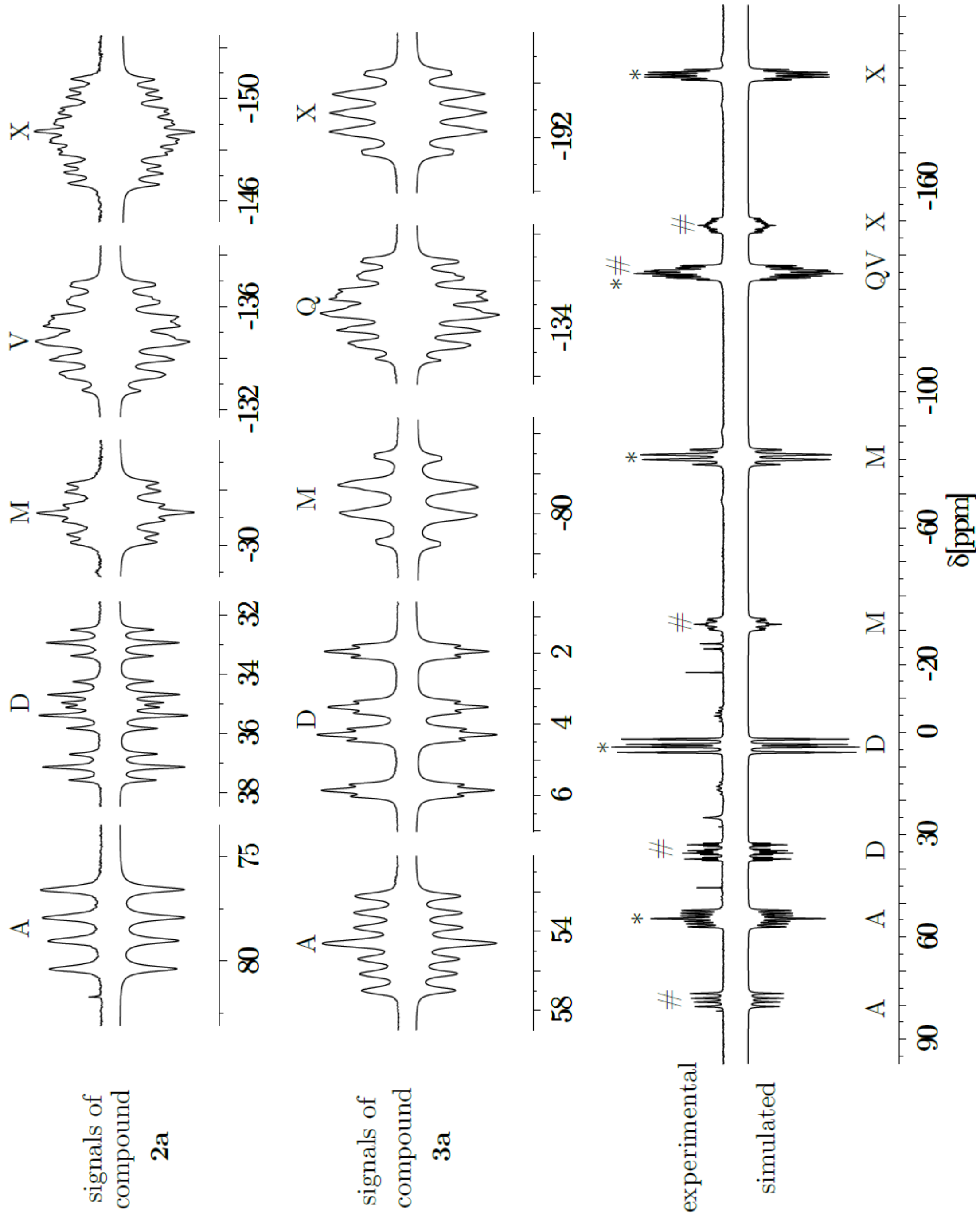

Figure S5: Simulation of the spin systems in the $^{31}P\{^{1}H\}$ NMR spectrum of compounds **2a** and **3**. The multiplets of compound **2a** are marked with ∗, the multiplets of compound **3a** with #. The multiplet Q/V is the result of two overlapping multiplets from the two different spin systems. The simulation of the spin system is shown inverted.

1.3 NMR spectra of reactions of **1** with [Me_2P][OTf] at 193 K

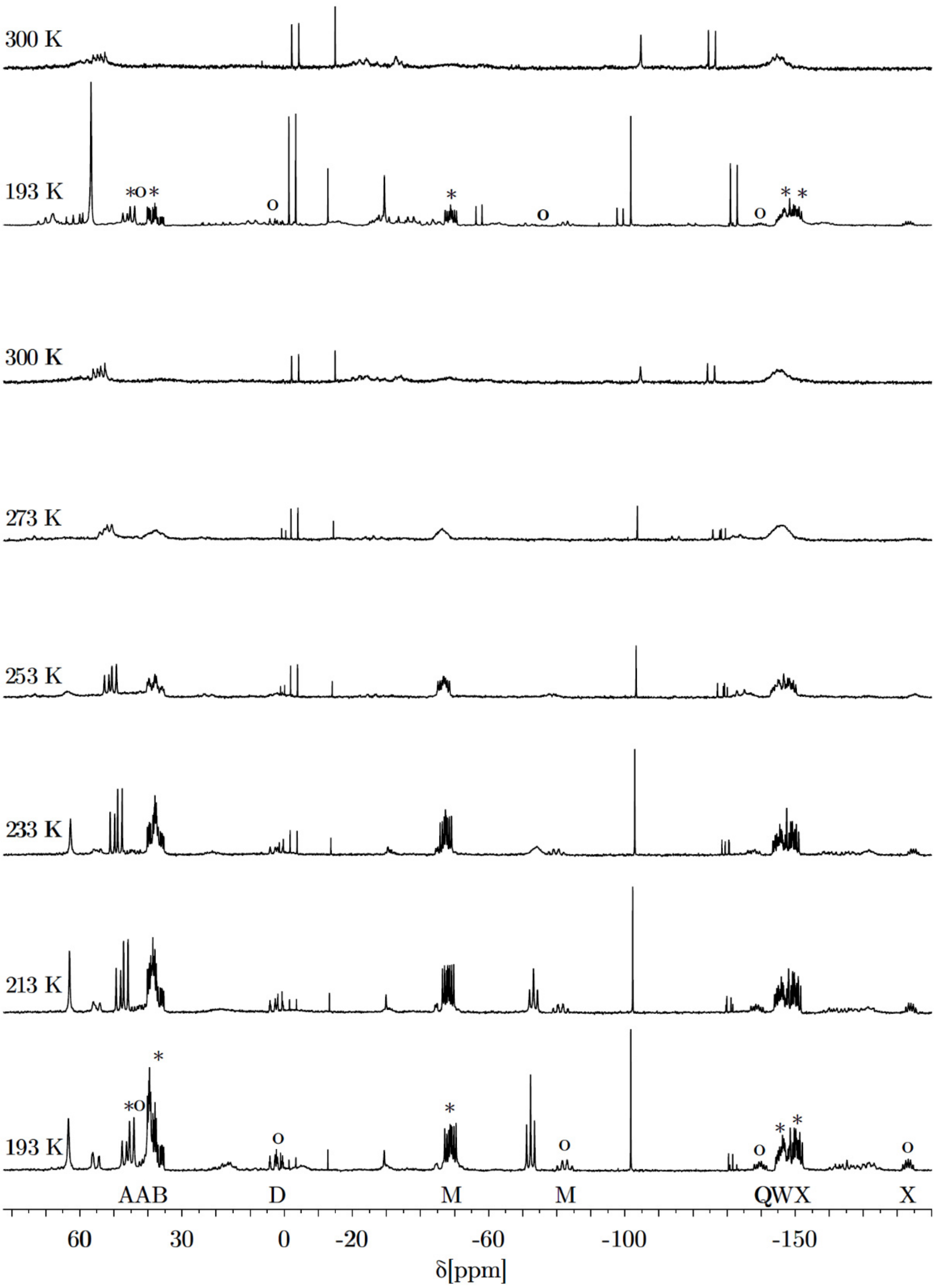

Figure S6: $^{31}P\{^1H\}$ VT NMR spectrum in CD_2Cl_2 of the reaction of **1** with in situ generated [Me_2P][OTf]. The signals of **2b** are marked with ∗, the signals of **3b** with o.

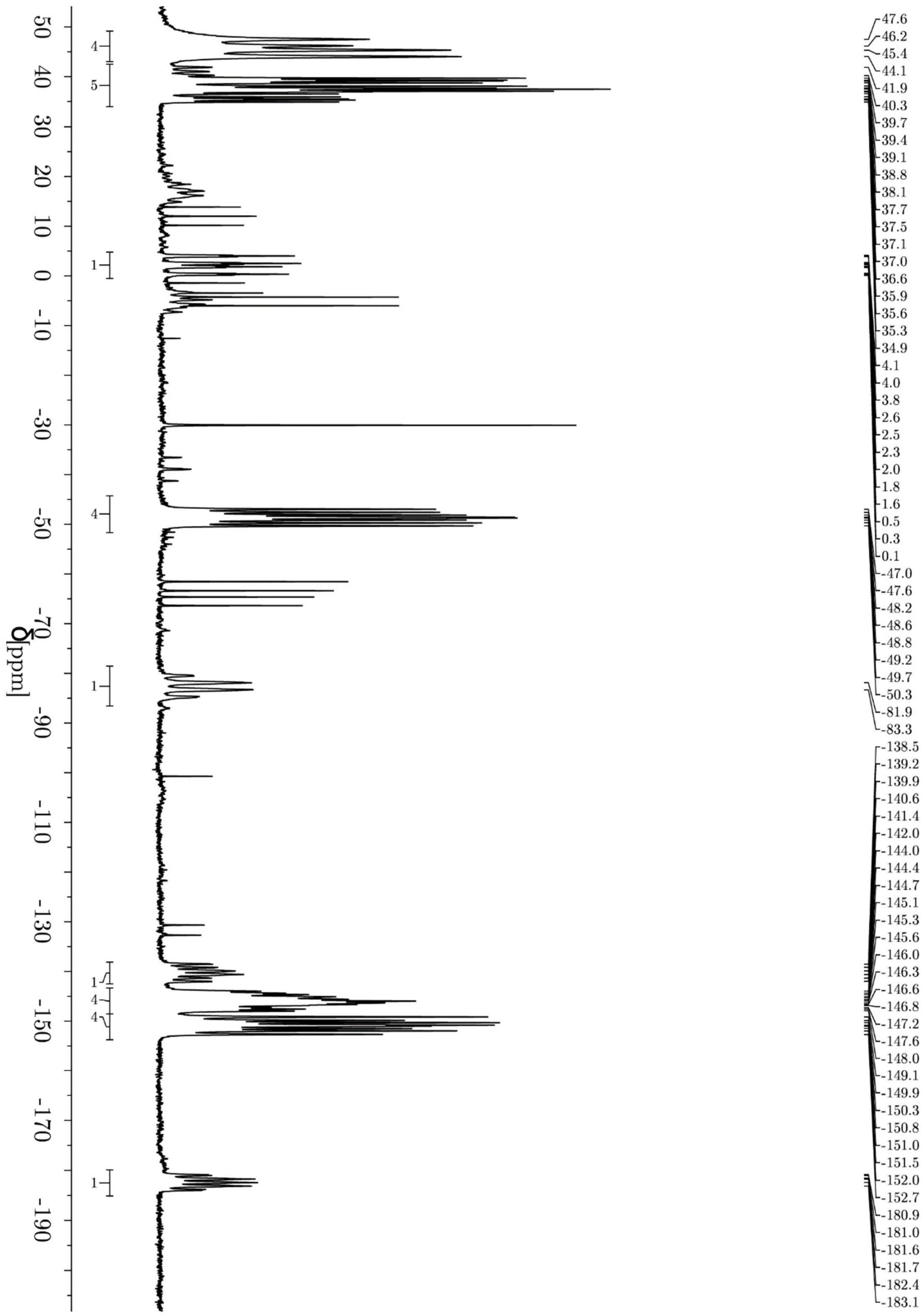

Figure S7: $^{31}P\{^1H\}$ NMR spectrum (excerpt) in CD_2Cl_2 from the reaction of **1** with $[Me_2P][OTf]$ at 193 K with chemical shifts and integrals depicted in the spectrum.

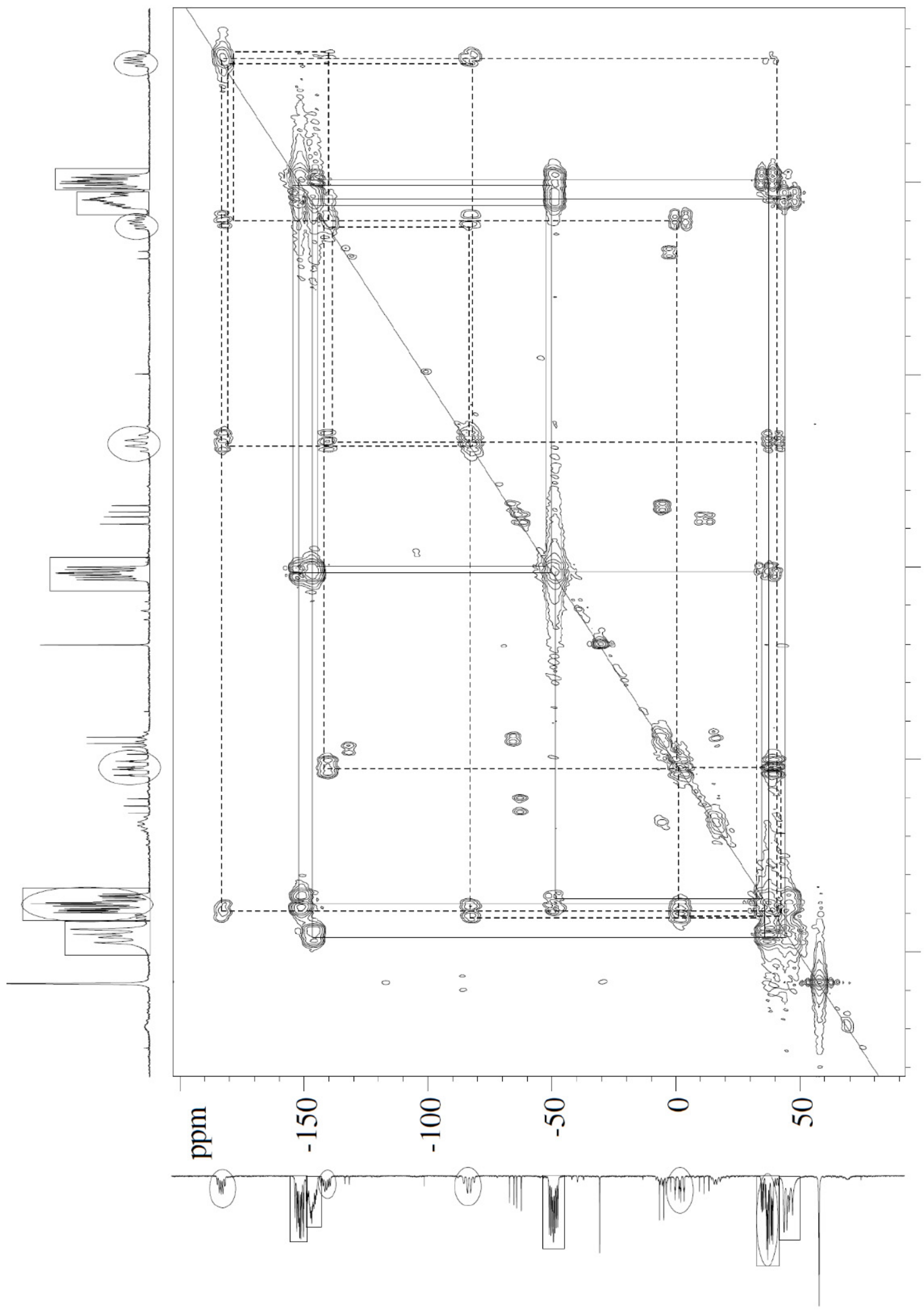

Figure S8: ^{31}P-^{31}P COSY NMR spectrum in CD_2Cl_2 at 193 K of the product mix from the reaction of $[Me_2P][OTf]$ with **1** at 193 K. Signals of **2b** are marked with rectangles, signals of **3b** with ellipses. Couplings in compound **2b** are drawn as rectangles with solid lines, couplings in compound **3b** with dashed lines. For easier understanding, the diagonal signals are connected via a solid line. The couplings between multiplets A and B as well as between W and X of compound **3b** are not resolved in this figure since they appear in close proximity to each other.

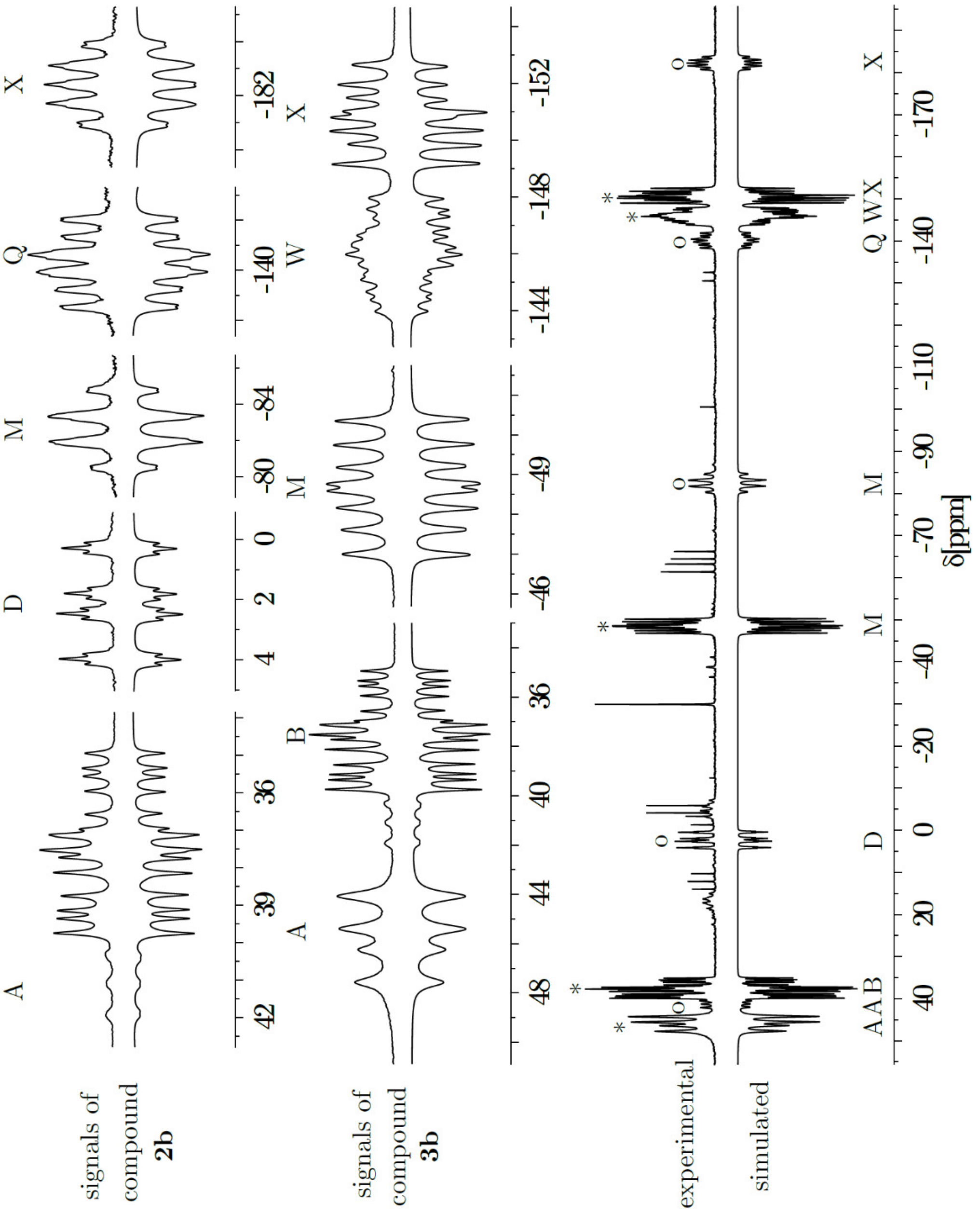

Figure S9: Simulation of the spin systems in the $^{31}P\{^{1}H\}$ NMR spectrum of compounds **2b** and **3b**. The multiplets of compound **2b** are marked with ∗, the multiplets of **3b** with o. The signal at 40 ppm is the result of overlapping multiplets B of compound **2b** and A of compound **3b**. The simulation of the spin system is shown inverted. The simulated multiplets W and X of **2b** differ from the signals in the real spectrum in intensity, which can be explained by overlaps with signals from minor products not accounted for in the iterative fit. The coupling pattern of the simulated multiplet does not differ from the real one.

1.4 NMR spectra of the reaction of **1** with in situ generated $[Ph_2P][PF_6]$ at room temperature

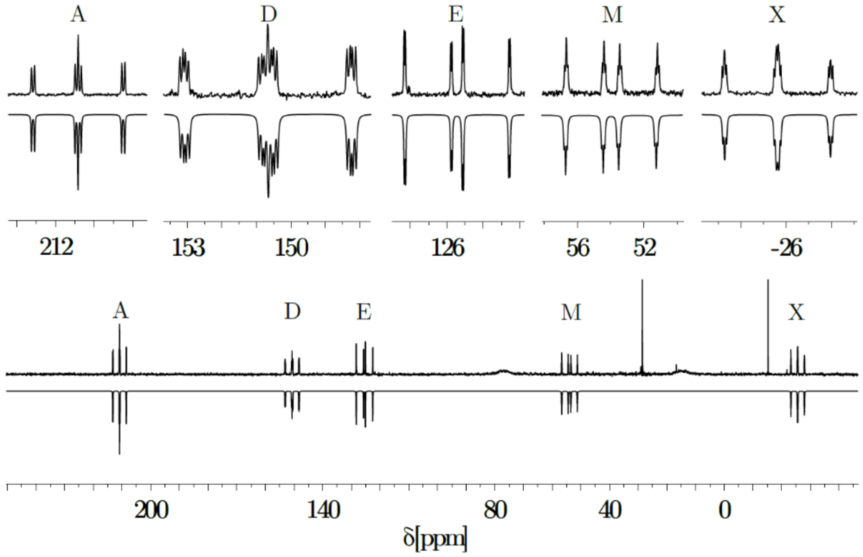

Figure S10: Simulation (inverted) of the $^{31}\{^1H\}$ NMR spectrum of **4**.

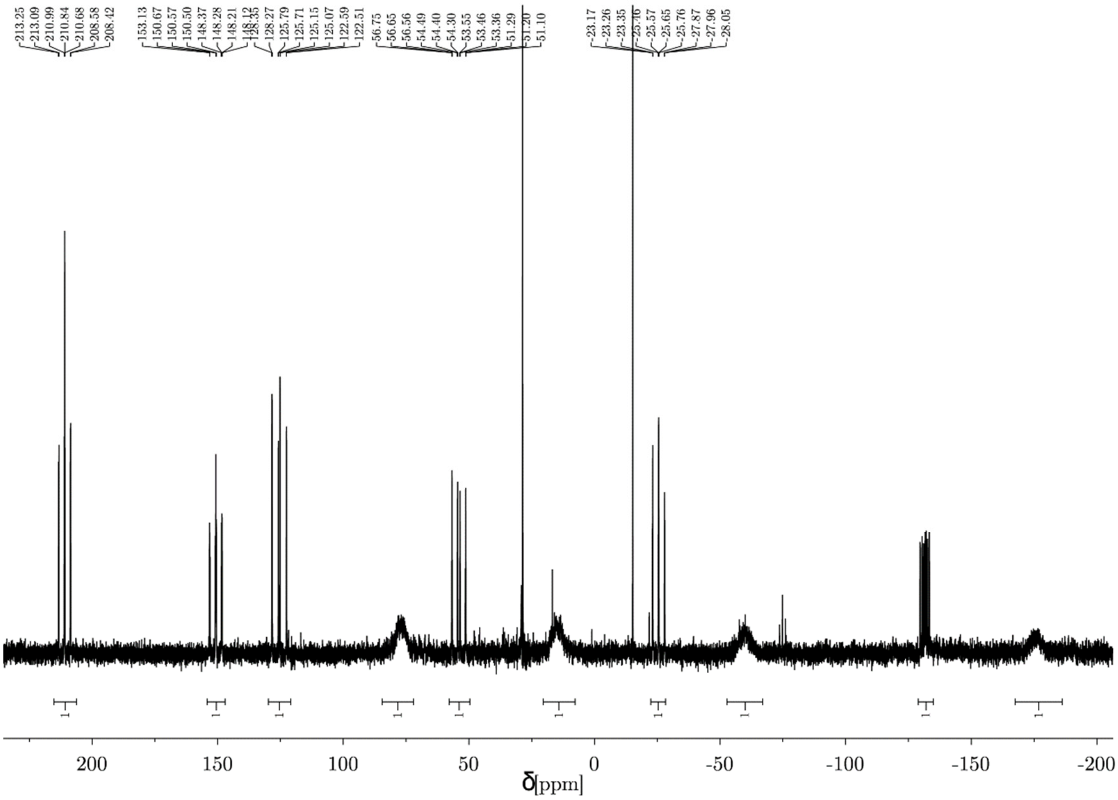

Figure S11: $^{31}P\{^1H\}$ NMR spectrum (excerpt) in CD_2Cl_2 from the reaction of **1** with $[Ph_2P][PF_6]$ at room temperature with chemical shifts and integrals depicted in the spectrum.

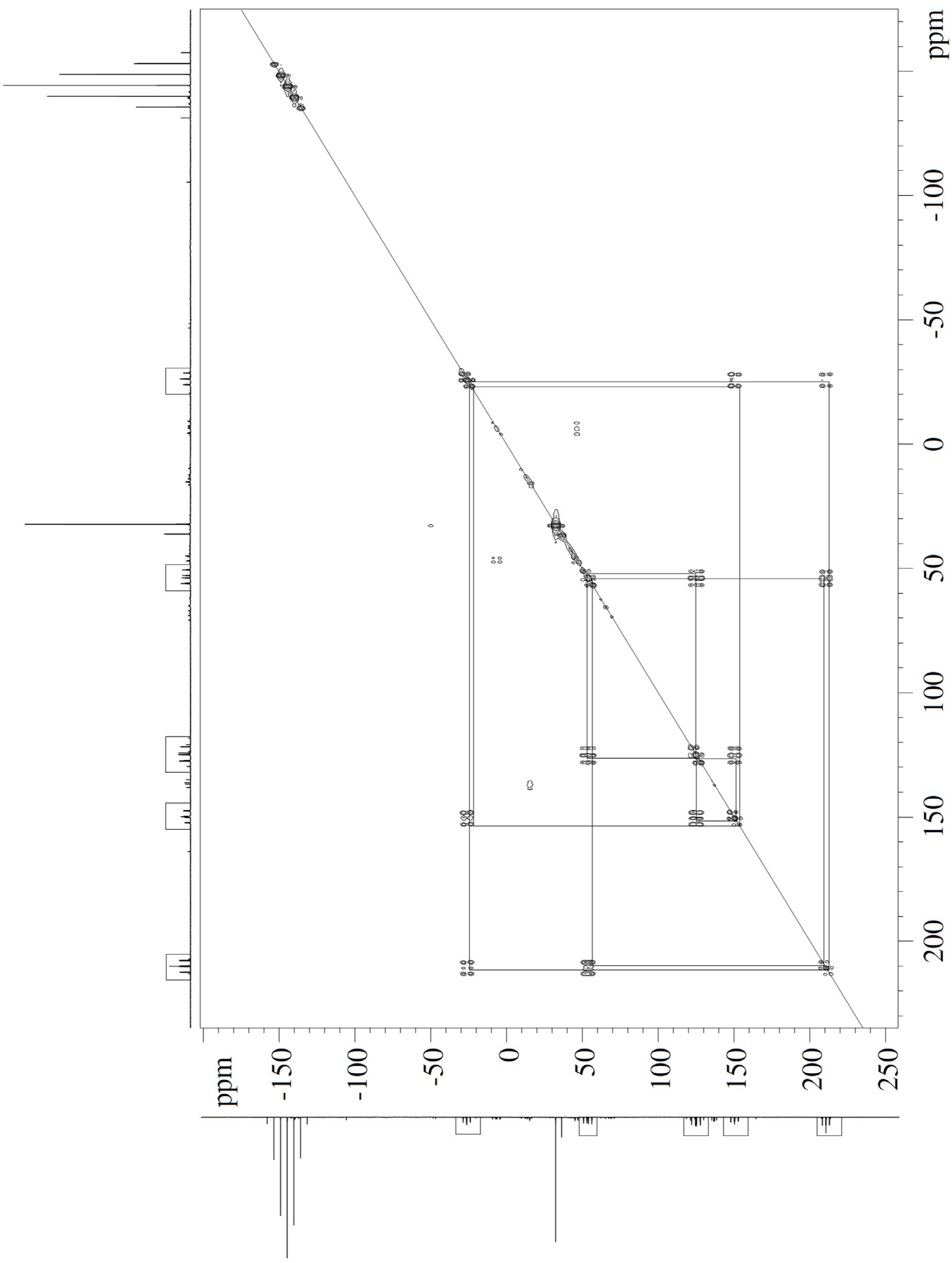

Figure S12: ^{31}P-^{31}P COSY NMR spectrum of **4** at room temperature. Couplings are marked with rectangles for easier understanding.

1.5 NMR spectra of the reaction of **1** with in situ generated [Ph_2As][OTf].

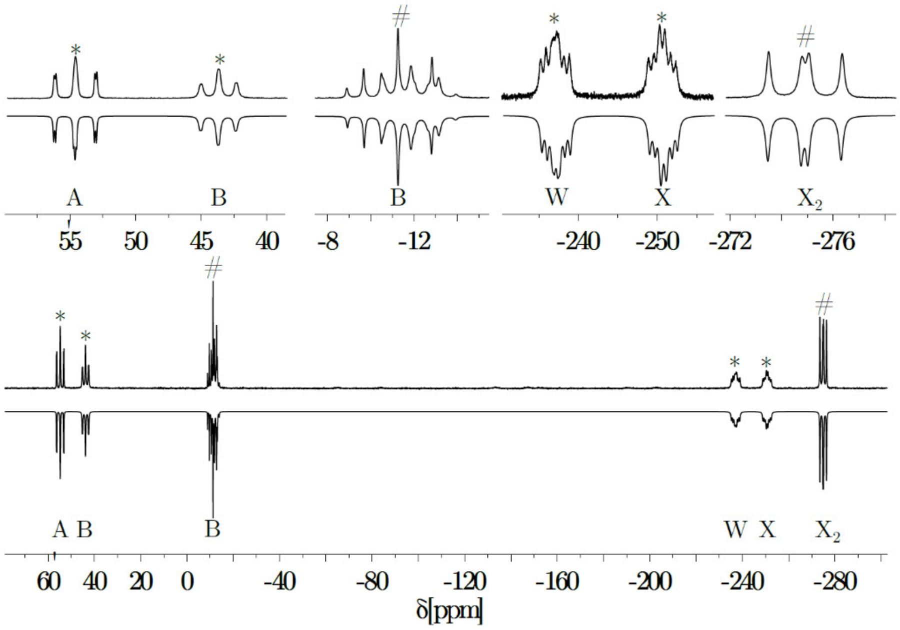

Figure S13: Simulation of the $^{31}P\{^{1}H\}$ NMR spectrum of coordination mode 1 and 2 of [Ph_2As][OTf] on **1**. Signals of coordination mode 1 are marked with ∗, signals of coordination mode 2 with #. The simulated spectrum is shown inverted.

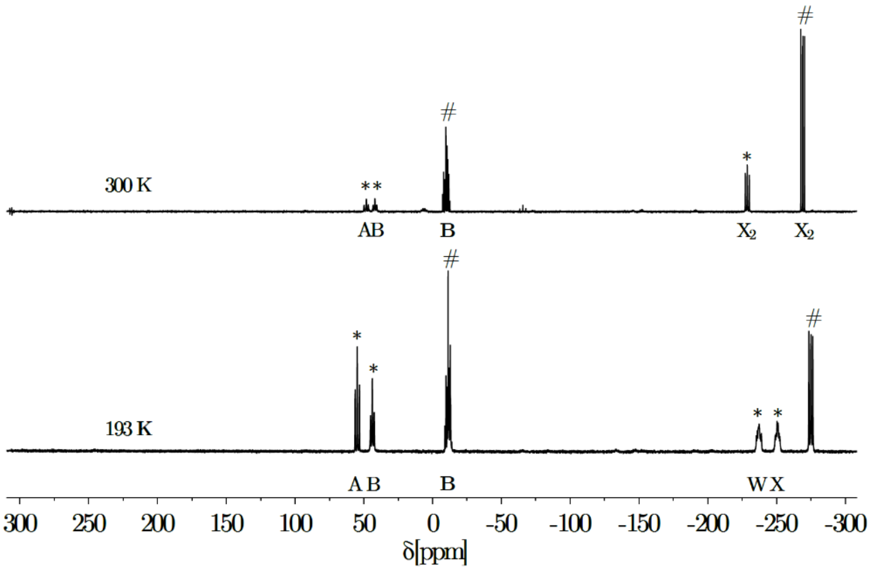

Figure S14: Comparison of the $^{31}P\{^{1}H\}$ NMR spectrum of coordination mode 1 and 2 of [Ph_2As][OTf] on **1** at 193 K and room temperature. Signals of coordination mode 1 are marked with ∗, signals of coordination mode 2 with #.

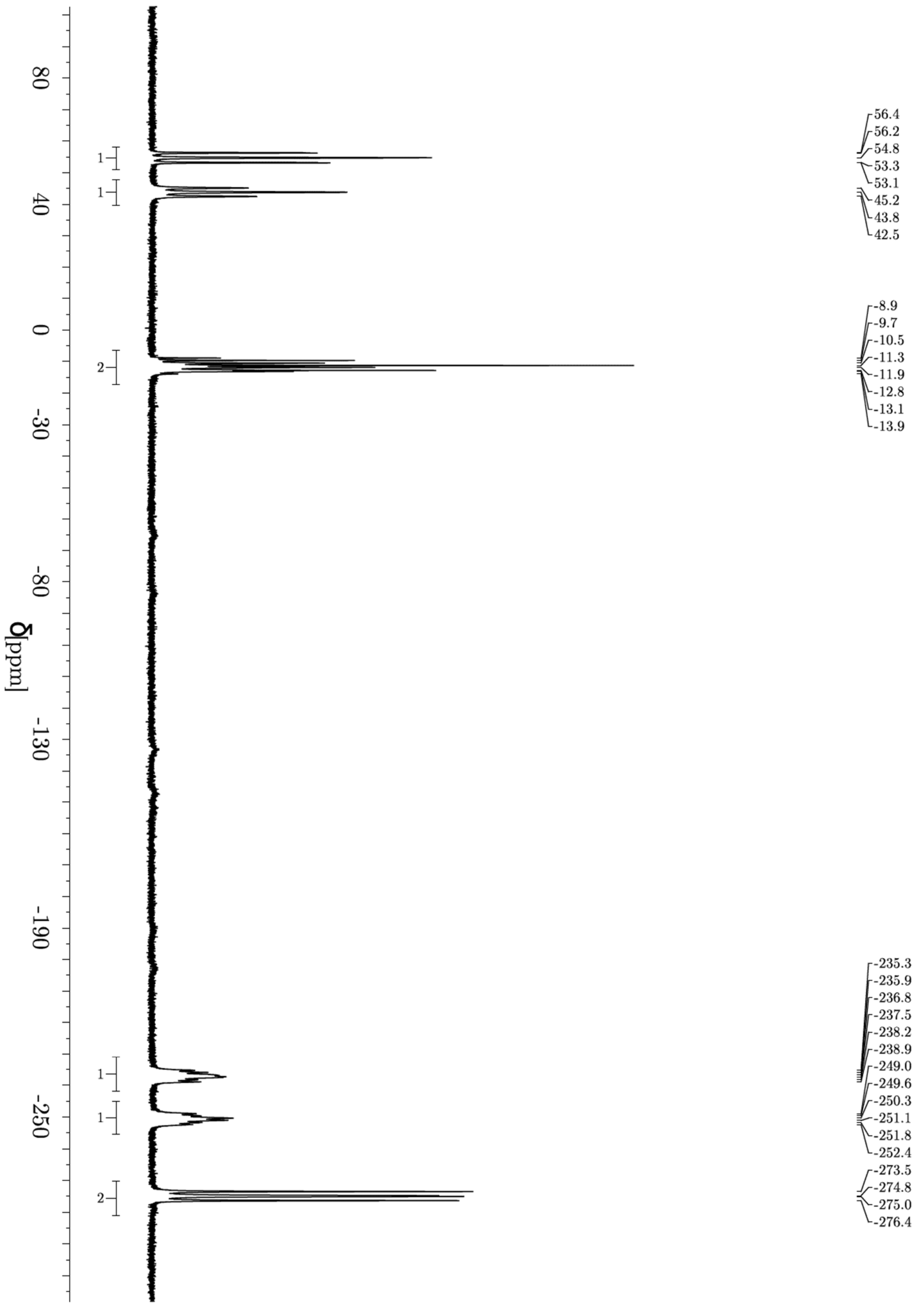

Figure S15: $^{31}P\{^{1}H\}$ NMR spectrum (excerpt) in CD_2Cl_2 right after warming to room temperature from the reaction of **1** with $[Ph_2As][OTf]$ at 193 K with chemical shifts and integrals depicted in the spectrum.

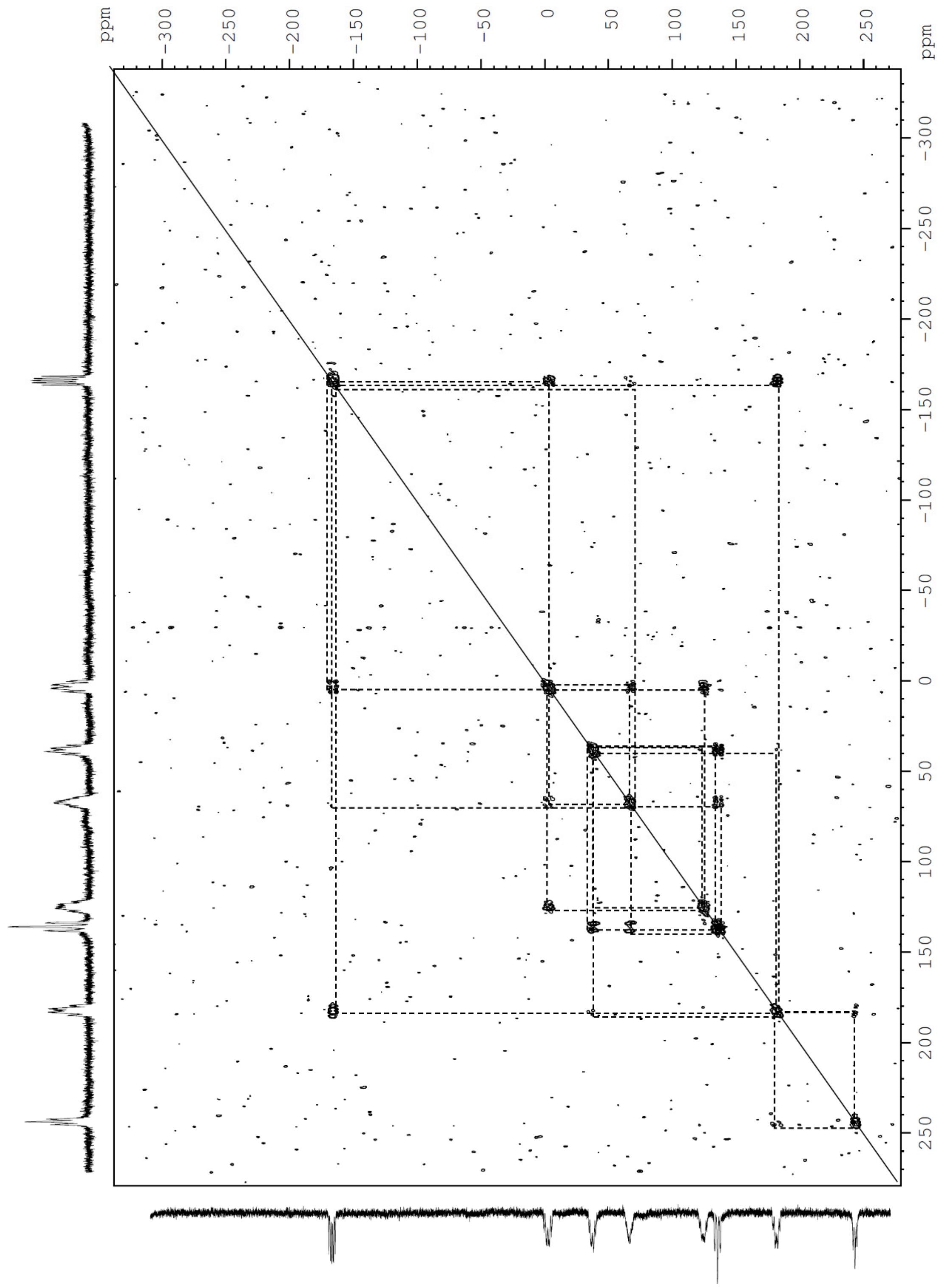

Figure S16: ^{31}P-^{31}P COSY NMR spectrum in CD_2Cl_2 at 273 K of **5**. For easier understanding, the diagonal signals are connected via a solid line and the couplings between multiplets are marked with dotted rectangles. Due to the dynamic behaviour some couplings are not or badly resolved.

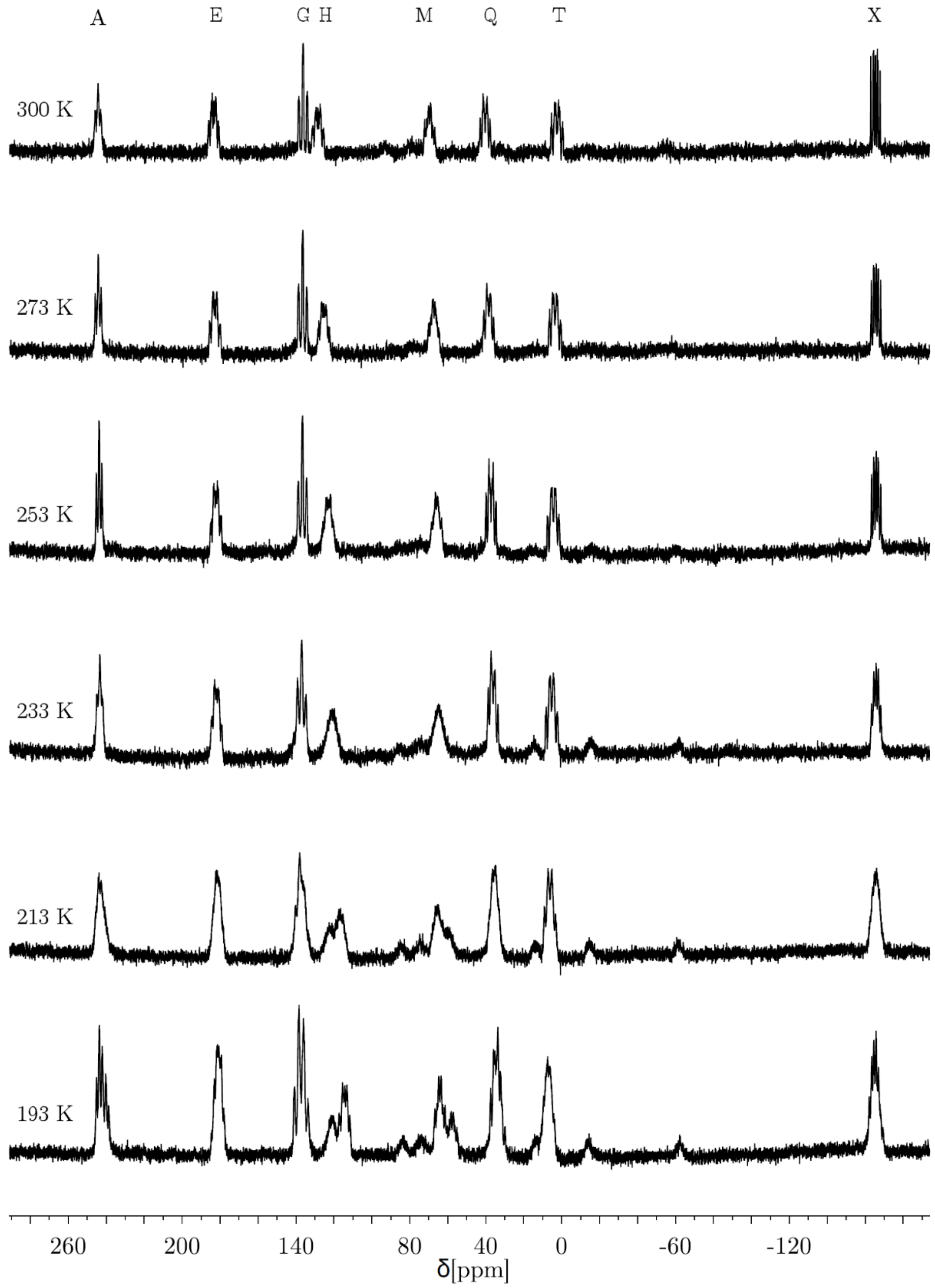

Figure S17: ^{31}P VT NMR spectrum in CD_2Cl_2 of crystals of **5**. The splitting of H and M as well as the beginning splitting of the other multiplets of the spin system can be explained that freezing dynamic processes, inhibiting free rotation of the [Cp'''Fe(CO)$_2$] fragments.

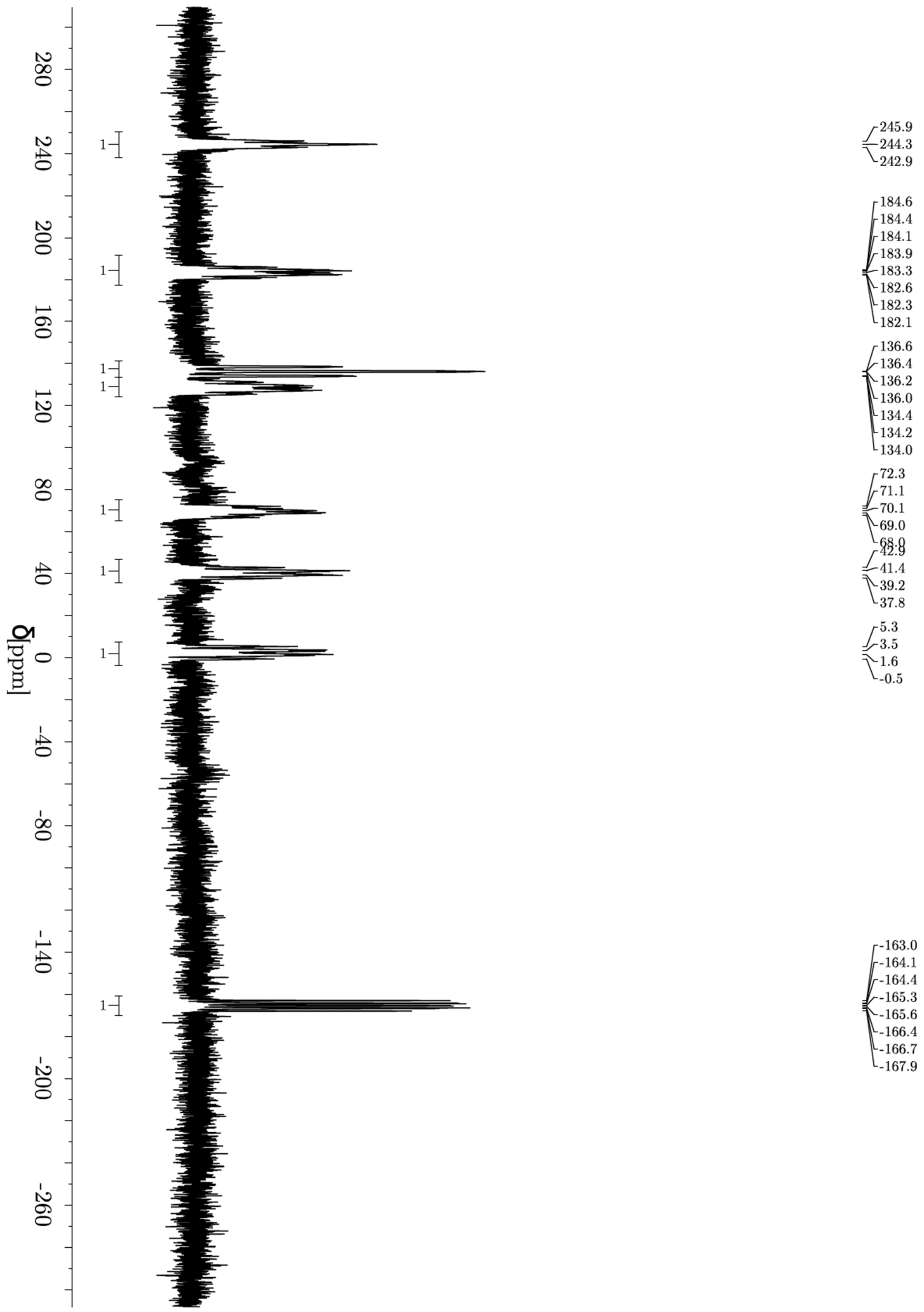

Figure S18: ^{31}P NMR spectrum of **5** in CD_2Cl_2 at room temperature with chemical shifts and integrals depicted in the spectrum.

Table S8: ^{31}P NMR data of **5**: (CD_2Cl_2; 273 K) with assignment of the individual phosphorus atoms:

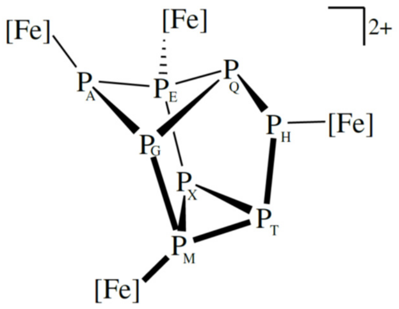

	δ (ppm)	Coupling with
P_A	244.3	P_E
P_E	183.3	P_A P_Q P_X
P_G	136.3	P_Q P_M
P_H	128.4	P_T P_Q
P_M	69.5	P_T P_G P_X
P_Q	40.3	P_E P_G P_H
P_T	2.5	P_H P_M P_X
P_X	-165.4	P_E P_M P_T

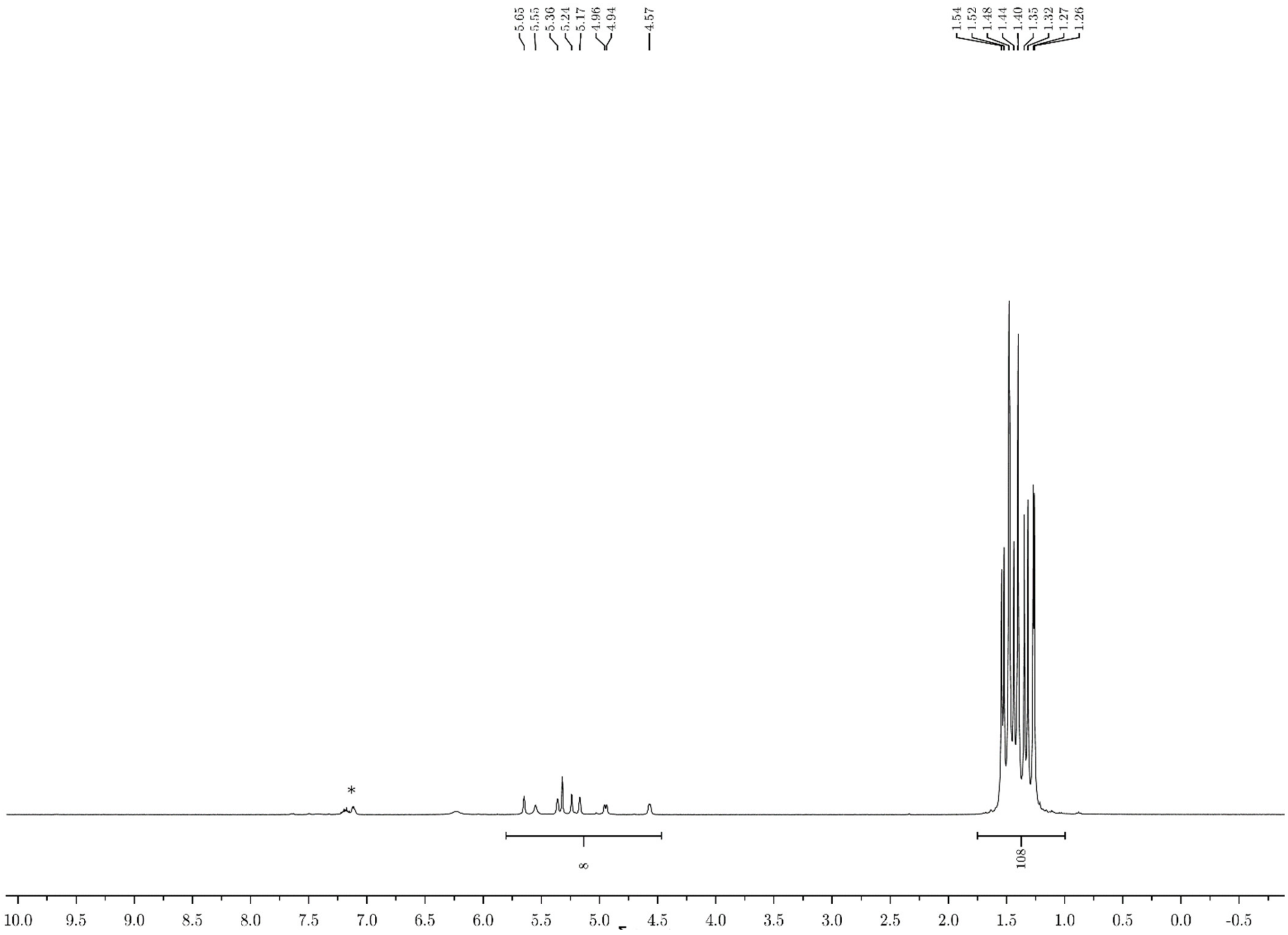

Figure S19: ^{1}H NMR spectrum of **5** in CD_2Cl_2 at room temperature with chemical shifts and integrals depicted in the spectrum. Signals from residual o-DFB are marked with ∗.

2 Crystallographic details

CIF files with comprehensive information on the details of the diffraction experiments and full tables of bond lengths and angles for **3a**, **4**, and **5** are deposited in Cambridge Crystallographic Data Centre under the deposition codes CCDC-2085799, CCDC-2085801, and CCDC-2085801, respectively.

2.1 Crystallographic details on **3a**

Compound **3a** was crystallized in very small amounts from an ortho-difluorobenzene pentane mixture by storing at 243 K for one month. A suitable clear red block- shaped crystal of **3a** with dimensions $0.07 \times 0.05 \times 0.04\ \mathrm{mm}^3$ was selected and mounted on a GV1000, TitanS2 diffractometer. The crystal was kept at a steady T = 123.00(10) K during data collection. The structure was solved with the ShelXT 2018/2 [1] solution program using iterative methods and by using Olex2 1.3-alpha [2] as the graphical interface. The model was refined with ShelXL 2018/3 [3] using full matrix least squares minimisation on F^2. Two of the tert-butyl groups on the Cp''' on Fe1 were disordered due to rotation.

2.2 Crystallographic details on **4**

Compound **4** was crystallized in very small amounts from a 1:1 toluene/diethylether mixture at 4 °C. The needle-shaped crystals of **4** were significantly undergrown, showing no scattering on the laboratory diffractometer. Therefore, we decided to perform the diffraction study using synchrotron radiation. The diffraction data for **4** were collected on the P11 beamline at PETRAIII accelerator at DESY, Hamburg, Germany [4]. The microscopic needle crystal was mounted on the 100 μm MiTeGen® loop in the drop of the mineral oil. The X-ray beam (E = 18 keV, λ = 0.6888 Å) was preliminary focused to $200 \times 200\ \mu\mathrm{m}$ with the focusing bendable KB mirror system [4] and then additionally collimated to 50 μm. As a result, the background was significantly reduced resulting in quite appropriate $\mathrm{I}_{\mathrm{hkl}}/\sigma$ values (average 5 before merging, 14 after the merging for the data up to 0.834 Å). Total 1800 frames were collected using 0.2° frames in the shutterless mode using Pilatus 6M detector. The CrysAlisPro software was used to extract the reflection intensities from the set of the frames. After the data finalization, the reflections up to $d_{min.}$ = 0.83 Å were used for the refinement. The structure was solved using SHELXT [1] program and refined in the anisotropic approximation using SHELX2018/3 [3] program. Both solvent molecules are disordered and therefore some restraints were applied in the refinement.

2.3 Crystallographic details on **5**

Compound **5** was crystallized from an ortho-difluorobenzene pentane mixture at room temperature. A suitable clear orange plate-shaped crystal of **5** with dimensions 0.26 × 0.17 × 0.13 mm^3 was selected and mounted on a GV1000, TitanS2 diffractometer. The crystal was kept at a steady T = 133.00(10) K during data collection, since we noticed loss of crystallinity at lower temperatures. The structure was solved with the ShelXT [1] solution program using dual methods and by using Olex2 1.5-alpha [2] as the graphical interface. The model was refined with ShelXL 2018/3 [3] using full matrix least squares minimisation on F^2. A solvent mask was calculated and 121 electrons were found in a volume of 493 $Å^3$ in 1 void per unit cell. This is consistent with the presence of 1[$C_6H_4F_2$] per asymmetric unit which account for 116 electrons per unit cell. One triflate anion was disordered.

2.4 Crystallographic data

Table S9: Crystallographic data for **3a**, **4** and **5**:

Compound	**3a**	**4**	**5**
Formula	$C_{50}H_{68}F_6Fe_2O_4P_6$	$C_{53.5}H_{77}F_6Fe_2O_{2.5}P_6$	$C_{96}H_{128}F_{12}Fe_4O_{14}P_8S_2$
$\rho_{calc.}/gcm^{-3}$	1.409	1.378	1.403
μ/mm^{-1}	6.525	0.700	6.400
Formula Weight	1144.56	1171.67	2269.26
Colour	clear red	black	orange
Shape	block-shaped	needle	plate-shaped
Size/mm^3	0.07 × 0.05 × 0.04	0.05 × 0.01 × 0.00	0.26 × 0.17 × 0.13
T/K	123.00(10)	100.00(10)	133.00(10)
Crystal System	triclinic	monoclinic	triclinic
Space Group	P-1	C2/c	P-1
a/Å	10.4574(5)	45.1781(12)	14.36190(10)
b/Å	13.7684(4)	9.86897(17)	15.92590(10)
c/Å	20.0423(11)	27.6331(5)	26.1504(2)
$\alpha/°$	99.992(3)	90	93.8200(10)
$\beta/°$	102.592(4)	113.564(2)	104.6430(10)
$\gamma/°$	100.656(3)	90	109.6900(10)
V/Å^3	2698.1(2)	11293.1(5)	5372.45(8)
Z	2	8	2
Z'	1	1	1
Wavelength/Å	1.54184	0.6888	1.54184
Radiation type	Cu Kα	synchrotron	Cu Kα
$\theta_{min}/°$	3.351	1.558	3.543
$\theta_{max}/°$	65.089	25.499	66.734
Measured Refl's.	14949	43651	105406
Indep't Refl's	8833	11357	18923
Refl's $I \geq 2\sigma(I)$	6565	6508	18211
R_{int}	0.0649	0.0773	0.0433
Parameters	691	703	1234
Restraints	0	94	48
Largest Peak	0.724	0.521	0.599
Deepest Hole	-0.895	-0.468	-0.552
GooF	1.006	0.797	1.057
wR2 (all data)	0.1469	0.0691	0.0936
wR2	0.1332	0.0665	0.0924
R1 (all data)	0.0783	0.0629	0.0368
R1	0.0562	0.0339	0.0356

2.5 Solid state structures

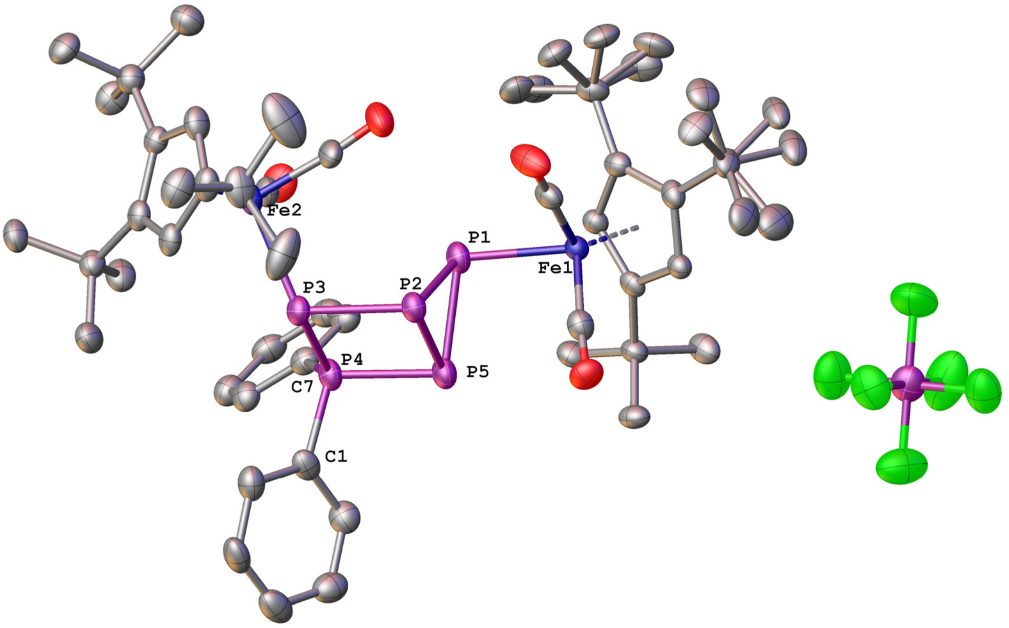

Figure S20: Molecular structure of **3a** in the solid state. Hydrogen atoms are omitted for clarity. Selected bond lengths and angles: Fe1-P1 2.3162(12), Fe2-P3 2.3004(15), P1-P5 2.2010(18), P2-P1 2.1971(15), P2-P5 2.2546(17), P3-P2 2.2305(15), P3-P4 2.2128(17), P4-P5 2.1974(16), P4-C1 1.813(5), P4-C7 1.819(4), P2-P3-Fe2 111.52(6), P4-P3-Fe2 124.22(6), P4-P3-P2 84.67(6), P3-P2-P5 94.38(6), P1-P2-P3 103.02(6), P1-P2-P5 59.24(5), P5-P4-P3 96.52(6), C1-P4-P3 107.66(16), C1-P4-P5 103.03(15), C1-P4-C7 104.1(2), C7-P4-P3 127.21(16), C7P4-P5 115.93(15), P2-P1-Fe1 101.85(5), P2-P1-P5 61.71(5), P5-P1-Fe1 101.19(6), P4-P5-P2 84.43(6), P4-P5-P1 97.58(7), P1-P5-P2 59.05(5).

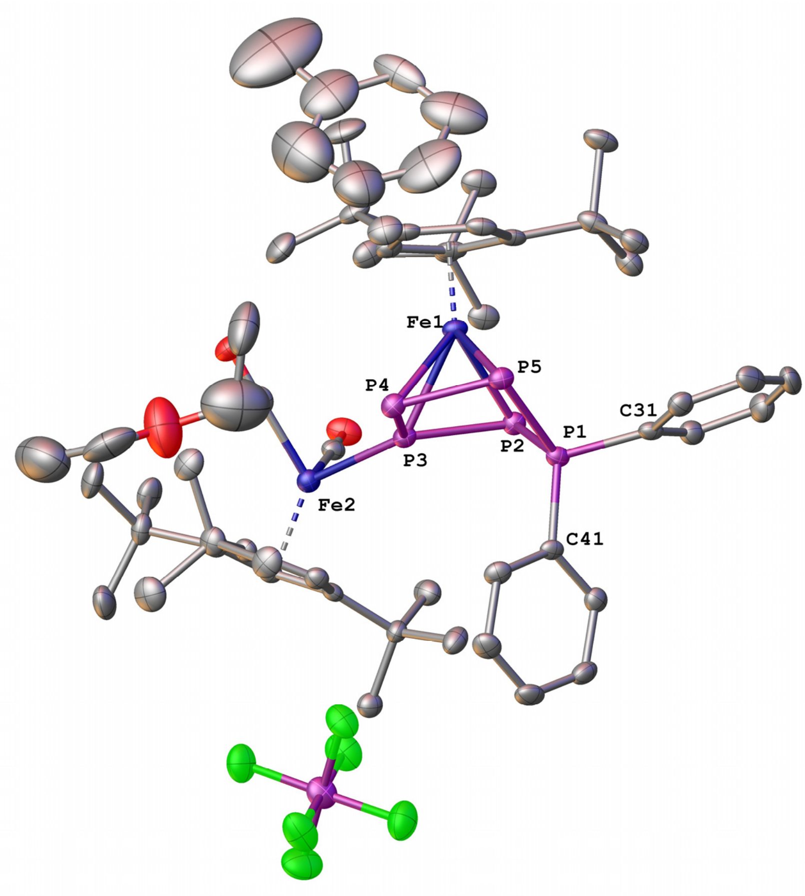

Figure S21: Molecular structure of **4** in the solid state. Hydrogen atoms are omitted for clarity. Selected bond lengths and angles: Fe1-P3 2.2925(7), Fe1-P5 2.3422(7), Fe1-P4 2.3517(8), Fe1-P2 2.3610(8), Fe2-P3 2.2567(7), P1-P5 2.1590(9), P1-P2 2.1761(9), P2-P3 2.1293(9), P3-P4 2.1337(9), P4-P5 2.1322(10), P3-Fe1-P2 54.44(2), P3-Fe1-P4 54.68(2), P3-Fe1-P5 90.91(3), P4-Fe1-P2 94.13(3), P5-Fe1-P2 86.41(3), P5-Fe1-P4 54.03(2), P5-P1-P2 95.93(4), C31-P1-P2 107.51(9), C31-P1-P5 106.08(9), C41-P1-P2 118.24(8), C41-P1-P5 119.46(9), C41-P1-C31 108.34(11), P1-P2-Fe1 88.26(3), P3-P2-Fe1 61.14(3), P3-P2-P1 95.50(4), Fe2-P3-Fe1 140.08(3), P2-P3-Fe1 64.42(3), P2-P3-Fe2 132.67(4), P2-P3-P4 108.06(4), P4-P3-Fe1 64.07(3), P4-P3-Fe2 119.16(4), P3-P4-Fe1 61.25(3), P5-P4-Fe1 62.76(3), P5-P4-P3 101.50(4), P1-P5-Fe1 89.16(3), P4-P5-Fe1 63.21(3), P4-P5-P1 99.70(4).

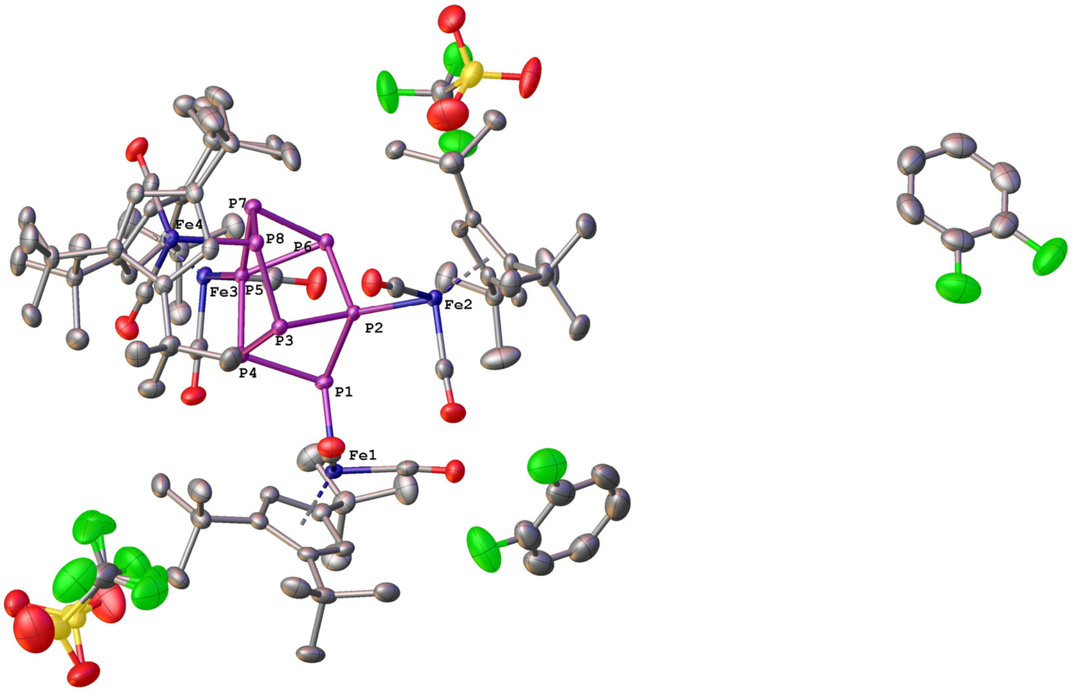

Figure S22: Molecular structure of **5** in the solid state. Hydrogen atoms are omitted for clarity. Selected bond lengths and angles: Fe4-P8 2.2756(5), Fe3-P5 2.2468(5), Fe2-P2 2.2587(5), Fe1-P1 2.3040(5), P5-P6 2.2115(7), P5-P7 2.1916(7), P5-P4 2.2134(7), P2-P3 2.1890(7), P2-P6 2.2713(6), P2-P1 2.2758(7), P3-P8 2.1612(7), P3-P4 2.2062(7), P6-P7 2.2071(6), P8-P7 2.1925(7), P4-P1 2.2402(7), P6P5-Fe3 122.66(3), P6-P5-P4 103.66(2), P7-P5-Fe3 130.27(3), P7-P5-P6 60.17(2), P7P5-P4 109.69(3), P4-P5-Fe3 116.00(2), Fe2-P2-P6 114.17(2), Fe2-P2-P1 132.07(2), P3-P2-Fe2 117.79(2), P3-P2-P6 102.17(2), P3-P2-P1 88.64(2), P6-P2-P1 96.02(2), P2-P3-P4 85.04(2), P8-P3-P2 100.83(3), P8-P3-P4 110.76(3), P5-P6-P2 95.91(2), P7-P6-P5 59.47(2), P7-P6-P2 104.70(3), P3-P8-Fe4 111.77(2), P3-P8-P7 100.87(3), P7-P8-Fe4 110.90(2), P5-P7-P6 60.36(2), P5-P7-P8 107.42(3), P8-P7-P6 103.88(2), P5-P4-P1 94.68(2), P3-P4-P5 98.62(3), P3-P4-P1 89.12(2), P2-P1-Fe1 117.51(2), P4-P1-Fe1 105.49(2), P4-P1-P2 82.26(2).

3 Details on DFT-calculations

3.1 General remarks

DFT Calculations were performed using the ORCA program package [5]. All calculations were performed in the gas phase and optimized and frequency calculations were performed on geometry optimisations to check for imaginary frequencies indicating local minima. Frequency calculation after geometry optimisation of **3a** showed one very small negative frequency at 5.9 cm^{-1} assigned to the rotation of one Cp''' fragment after multiple optimisation attempts and therefore was ignored. The relative energies of **1**, **2a**, **3a**, the decarbonlyated form of **3a** and **4** along with all reagents involved were calculated on the BP86 [6–8] /def2-SVP [9,10] level. Input files were generated from solid state structures if possible. Avogadro was used as the graphical user interface [11].

3.2 Thermodynamic data from frequency calculations

Table S10: **1**, **2a**, **3a**, the decarbonlyated form of **3a**, **4**, CO and $[Ph_2P]^+$ (T = 298,15 K; Energies are given in Hartree, S in Hartree/K, E_{SCF} is the total SCF energy):

Compound	E_{SCF}	H	S	G
**1**	$-5.6744393\cdot10^3$	$-5.6745817\cdot10^3$	$4.81382458\cdot10^{-4}$	$-5.67472523\cdot10^3$
**2a**	$-6.4795590\cdot10^3$	$-6.4784465\cdot10^3$	$5.59401308\cdot10^{-4}$	$-6.47861331\cdot10^3$
**3a**	$-6.4782691\cdot10^3$	$-6.4784588\cdot10^3$	$5.52270501\cdot10^{-4}$	$-6.47862354\cdot10^3$
**3a**–CO	$-6.3662735\cdot10^3$	$-6.3651717\cdot10^3$	$5.40210833\cdot10^{-4}$	$-6.36533285\cdot10^3$
**4**	$-6.2530463\cdot10^3$	$-6.2519551\cdot10^3$	$5.24493174\cdot10^{-4}$	$-6.25211153\cdot10^3$
CO	$-1.1322541\cdot10^2$	$-1.1321765\cdot10^2$	$7.53027335\cdot10^{-5}$	$-1.13240104\cdot10^2$
$[Ph_2P]^+$	$-8.0396198\cdot10^2$	$-8.0378271\cdot10^2$	$1.62933992\cdot10^{-4}$	$-8.03831294\cdot10^2$

3.3 Cartesian coordinates of optimised structures

Cartesian coordinates for **1**

Fe 3.46707827004776	5.83255580306058	4.76552523561312
Fe 9.33205094794833	9.95471874186724	3.65058297647087
P 4.82821982199349	7.56432261214437	3.92636772528074
P 6.98304365452824	6.89857327931471	3.74085139956528
P 6.41713451293886	8.23198822404679	5.41704754611118
P 7.13405175462827	9.15505490285786	3.48034492883929
O 5.43686405330763	5.09768179620043	6.78389482639981

O	4.64739429040817	3.97760303240448	2.85208539983040
O	8.59463360093019	11.55604418182907	5.97177403638688
O	10.08312534190273	7.68141357154358	5.31344590990229
C	1.75972621241572	5.38462354505798	5.96616679330905
C	1.97731044978123	6.85247411672182	5.96415873272510
C	1.88518614956429	7.26469727251698	4.58537973110867
C	1.60830383368218	6.16008496819697	3.72509267634951
C	1.54427064943468	5.01204944557006	4.59292582757097
C	10.69172395233700	9.47761630485248	2.04628381888130
C	11.22630091101673	10.59151212541045	2.85799281386399
C	10.23651535793696	11.63692564629830	2.78147293939379
C	9.13266799721689	11.26286524476249	1.93932197068116
C	9.42389624871807	9.92879708190272	1.51775369800801
C	1.51290488401715	4.31859614702052	7.06305480494812
C	-0.01781836005398	4.29633984467156	7.33213441666073
C	2.25466793395623	4.52913418481932	8.39942300627070
C	1.93056945649511	2.91550574119002	6.54745778548596
C	4.19107318427659	4.74365209598225	3.60649899385280
C	4.65619515867948	5.43203791067793	5.98249480833576
C	8.85375213100492	10.90152776297255	5.04078938099886
C	9.76364368780446	8.57520900126706	4.63371340047387
C	2.10894029978755	7.90617700334577	7.09270464182215
C	0.83860905996682	7.86699466487775	7.98185301090749
C	3.38309937256180	7.73757500719080	7.95222875222434
C	2.17986530260463	9.33157205079824	6.48561066363732
C	1.23306708742998	6.22313811896751	2.24346441555480
C	-0.16583133159493	6.89341333045907	2.16164313394887
C	2.24806012894747	7.06127229284180	1.43626946920056
C	1.14406714705048	4.80726320541628	1.63389737959565
C	11.28069057514398	8.14830756794409	1.51186831947524
C	12.01248588991017	8.48498912358574	0.18247167191640
C	12.25805515275884	7.41043803951217	2.45111854567352
C	10.13768401768110	7.14962634692831	1.18977344553939
C	12.57602810144450	10.83320402901804	3.57930593742151
C	13.73278812043468	10.73768611152832	2.55238649361690
C	12.61962527479949	12.26888080614346	4.16251504767968
C	12.82014252764407	9.87617695761061	4.76917527540661
C	8.02119527218459	12.19114332818658	1.44472154739870

C	8.69191394063063	13.27326747512046	0.55643495433757
C	7.29766864088352	12.87763490779248	2.62522592190753
C	6.99129295296075	11.41506145260804	0.59733360458798
H	2.02035538278442	8.29503725182757	4.24107782547994
H	1.34651765585157	3.99100651122922	4.25297219156670
H	10.32308489975553	12.60725724843680	3.28057402267174
H	8.77601710304090	9.32496483674490	0.87518095611553
H	-0.58598345645635	4.07960010533362	6.40461961954534
H	-0.37998967444559	5.26536196863209	7.72713444971145
H	-0.26329269477831	3.51094526250431	8.07757961137690
H	1.99585545161762	3.69990388273630	9.08977026485194
H	1.96971157740024	5.46940029097200	8.90569298682104
H	3.35355193812042	4.52189061973758	8.26433477010797
H	1.31237437033780	2.56759933608668	5.69659923711691
H	1.80423058726294	2.17017159124495	7.35887694698044
H	2.99221422113581	2.90447138898197	6.23069334907500
H	0.74708872701137	6.92839957836682	8.56034891621296
H	-0.08080185378215	7.97883961894429	7.37024249224677
H	0.86408480864385	8.70242301410079	8.71222022491248
H	3.39288556027809	8.48863890332653	8.76963444294316
H	4.28758733210304	7.89981903902760	7.33266097686595
H	3.46603355957740	6.73967056277335	8.41247163106923
H	2.28230497582482	10.07063007444876	7.30616793710545
H	1.26367205347426	9.59466457218142	5.91749857688390
H	3.05826572655074	9.45570947859599	5.82003381425070
H	-0.51096531720805	6.93913219085956	1.10763786321080
H	-0.13956353722689	7.92888813094469	2.55666324576181
H	-0.91875696071912	6.32469196501465	2.74498469433026
H	3.24448294808247	6.57715399644605	1.41877454361333
H	2.38409763491514	8.07682198156960	1.85911057862105
H	1.90053831970810	7.17632887927104	0.38856308649558
H	0.88643287175268	4.87461984791629	0.55725184245512
H	0.35830949323681	4.19738453898974	2.12495879659444
H	2.10808022459696	4.26740826082314	1.71671965457037
H	12.87113373210825	9.16483598914802	0.34428718538648
H	11.32618259677543	8.97328418885304	-0.53910790778846
H	12.39573500123542	7.55467873718596	-0.28605926571768
H	13.17170204061032	7.99231375974435	2.66799021523124

H 12.58331806384829	6.46896852191910	1.96398058718662
H 11.78066827605907	7.13571047538196	3.41134319505434
H 10.57308337635641	6.17748735140047	0.88370910739141
H 9.49450327789576	7.48879726453334	0.35384118128655
H 9.48749801762101	6.97359477287645	2.06999075928021
H 14.69218311839327	11.02001712397613	3.03328649010750
H 13.56670014737877	11.42592214450076	1.69826653948901
H 13.85414348090408	9.71773632885737	2.14629179400305
H 13.58970478134708	12.42339841610084	4.67676451016195
H 11.82165828865059	12.43712487354585	4.91348958450124
H 12.53369917498405	13.04604285625540	3.37598910739340
H 12.75976713438804	8.81163173480157	4.48977132873082
H 12.07297574462517	10.05462224297802	5.56732533605726
H 13.82824651749585	10.05761865587989	5.19889159948996
H 9.23264451227385	12.81553695406731	-0.29752281100224
H 9.41887425209552	13.88377966290610	1.13098365139148
H 7.92372498837783	13.96042055338324	0.14395100825146
H 6.55614796001459	13.61328984104542	2.24897308532580
H 8.00377372578613	13.42438449632983	3.28370837328723
H 6.75786998311212	12.13341218305217	3.24504977315318
H 7.45740102757104	10.96581766937958	-0.30389569101747
H 6.19300012386769	12.10168469219501	0.24789193408914
H 6.51560310762961	10.60482717659705	1.18722196454130

Cartesian coordinates for **2a**

Fe 3.43103480027236	6.27653491873993	4.22122356596875
Fe 9.44125697807932	10.11913957509240	3.62384711103469
P 4.56087226051688	8.13131354006404	3.40573316732215
P 6.74004640390710	7.62402880155145	3.07528422440876
P 6.14748099088583	8.95463961996078	4.77991099813488
P 7.23396797725428	9.84353187723092	2.99407759436126
O 5.74629999994932	5.48041179990348	5.81901997811696
O 4.41179947206707	4.65992619126567	1.99078688582499
O 8.63875675751899	11.76411700521482	5.89884826063651
O 9.52843561592740	7.68450122855279	5.23250049086745
C 2.22602548546462	5.46476839724110	5.82263884668119
C 2.12116906791885	6.94266407368171	5.81832533322243
C 1.57723242751476	7.29841861056179	4.52333203960959

C 1.30853806302225	6.13728658936456	3.73363698056255
C 1.77472213631231	5.03033155253414	4.52532272843292
C 11.01114543072608	9.45501496158686	2.29695381915431
C 11.54636808942367	10.43213188267729	3.27001093968568
C 10.78214103973571	11.63845912889292	3.06610604667307
C 9.83117007485840	11.49447432086660	1.99911274659786
C 9.97128630971882	10.13969873710549	1.56038589644288
C 2.43850323256122	4.37517422732114	6.90658895447245
C 1.00555296191329	4.02716682966231	7.41220932094519
C 3.31382334906211	4.73759855042628	8.12399682982595
C 3.04732350693861	3.08969375048084	6.28424692078662
C 4.00074244622587	5.32967353310077	2.84703981843808
C 4.84680430161142	5.85029419488300	5.18406121840497
C 8.93048980388848	11.10408357536747	4.98469619465009
C 9.48776027376164	8.64329254081378	4.57102929916533
C 2.27700799851183	7.99978840797371	6.93570477048651
C 1.21294622189551	7.71479540991639	8.03080636198386
C 3.69291408533477	8.05055945196674	7.55273310403552
C 1.97906465533086	9.41382576903332	6.38165600704456
C 0.42810722772883	6.00558593518067	2.49144879604764
C -0.93721564834960	5.47180115519720	3.01606082647130
C 0.19772972322138	7.36374479479193	1.80703010645984
C 1.00183060429177	5.00311721184299	1.46791947640632
C 11.47981643717554	8.06920345068049	1.79060899801108
C 12.47691921436214	8.35091245095660	0.62934465716600
C 12.16122648885010	7.15239186685429	2.82759765856610
C 10.28551648113706	7.26883787486498	1.21006851878079
C 12.74430217789690	10.41794150859940	4.25383872810959
C 14.05481629788708	10.19497762980991	3.45288859233754
C 12.87929267374302	11.79764620335348	4.94863592108316
C 12.61173645208715	9.37328868434279	5.38735828950073
C 9.03912564712142	12.62735274715373	1.34434412644372
C 10.07723975731132	13.54869392964141	0.64596717213403
C 8.25792546335351	13.44713897866547	2.39545477283552
C 8.06114245017773	12.08476381987266	0.28090822528167
H 1.37487087919809	8.32583167492497	4.20342028511442
H 1.72279364500403	3.98204297544477	4.21283751123087
H 10.93201432354375	12.56285587767455	3.63301399841508

H 9.38977334801091	9.68427446352273	0.75246466790113
H 0.35645094212247	3.67334417545788	6.58596007803908
H 0.50904970931466	4.89581326807390	7.88641284439197
H 1.06597402985737	3.21689555562110	8.16710844741066
H 3.36074900364963	3.85811997090997	8.79697930004868
H 2.90240289557686	5.57096091503135	8.72045826233907
H 4.35380980924249	4.98271531429173	7.83826156199103
H 2.39852009157699	2.62785738698390	5.51518576951245
H 3.17640799359774	2.33115845241338	7.08131891617290
H 4.04409612216832	3.27371834125675	5.83771991514564
H 1.39167946216915	6.76202812061228	8.56241173858276
H 0.19149394620960	7.68112629086620	7.59954358457888
H 1.23241940908356	8.52527310154457	8.78753808360903
H 3.71514021573731	8.79481340674042	8.37459065485336
H 4.43845357669952	8.36747667825052	6.79640690393689
H 4.02460740942974	7.08541454007375	7.96724551285326
H 2.12452973824047	10.15748898914586	7.18982420076111
H 0.93440174726062	9.51857132979711	6.02693167220172
H 2.65998628838832	9.69403480428249	5.55320138787248
H -1.65495390565630	5.38993210339649	2.17443748540670
H -1.37581674343576	6.15300161021888	3.77340710094123
H -0.83782287601389	4.46719525349044	3.47481171582343
H 1.14918904432056	7.80655943269299	1.45373527110776
H -0.30484853608628	8.08195175079295	2.48633122043678
H -0.45829023141258	7.23477074010024	0.92315307073336
H 0.26239595064603	4.82714363718351	0.66084020259713
H 1.23155233817468	4.01783745257609	1.92154335577730
H 1.92384804758680	5.39427459616162	0.99631433827555
H 13.37035939052943	8.90518423172002	0.97576452511862
H 12.00076300847407	8.94586384154482	-0.17616324650344
H 12.82079751771098	7.39291949514392	0.18790515539744
H 13.09107925898501	7.58261206553142	3.24009450285238
H 12.44021642819557	6.19961354351041	2.33402154382194
H 11.48538875844271	6.90161611244284	3.66763501734475
H 10.63964765948275	6.27250394038968	0.87820480699960
H 9.82735823241388	7.75312929447331	0.32552675924200
H 9.49245454885784	7.10853499133043	1.96842214914230
H 14.92819482344987	10.30259827613476	4.12793796942167

H 14.16217517605191	10.94366004862021	2.64146975418267
H 14.11348358358132	9.18880051976041	2.99788685264438
H 13.73481003006717	11.76695412266050	5.65228568859861
H 11.97951167118477	12.05702595079877	5.54254650776653
H 13.07819063049477	12.61678316636676	4.22836167591524
H 12.46444109411515	8.34463985013433	5.01969291701068
H 11.75947953736619	9.62256145617508	6.05014566394634
H 13.53163268027935	9.37973149965043	6.00736315949867
H 10.67563465701716	12.98797985566118	-0.10075303325216
H 10.77921412001335	14.00276348178456	1.37465087530728
H 9.55863139305021	14.37424858242818	0.11632460619565
H 7.75825342142272	14.31112366173096	1.91158008063861
H 8.92062261616328	13.84975508605015	3.18821638157268
H 7.47680493378325	12.82882780728919	2.88276319757959
H 8.59302713782558	11.54959514770107	-0.53223310883845
H 7.50792551450303	12.92473997152439	-0.18595041362974
H 7.31446668931123	11.39385925454224	0.72423746364264
P 4.14170673665717	9.83731953860904	1.96267039218050
C 2.89335615325153	10.87275303510507	2.84386413890554
C 3.44115711869234	11.88163707429697	3.67489266953831
C 2.60398645011460	12.77548746111229	4.36104526440232
H 3.04495457773025	13.55479276713573	5.00128600024333
C 1.20885011804230	12.68653333853819	4.21506067838764
H 0.55190040536160	13.39586785358161	4.74128277739651
C 0.65484989263055	11.69448343026586	3.38579241833535
H -0.43727760415453	11.62643485022103	3.26263672394948
H 4.53531152179849	11.96831778942984	3.77390109979093
C 1.48793008056452	10.78934366707325	2.70802151886432
H 1.04232478459846	10.02280844628166	2.05907730205358
C 3.30758784327981	9.06271597068704	0.51849242661061
C 2.44275504121830	9.83492645570127	-0.29749984810060
H 2.14417665064110	10.84826337823548	0.01094656905430
C 1.97258760346017	9.32441355199917	-1.51832061189396
H 1.29884199820329	9.93743927033893	-2.13669274285747
C 2.36347755983834	8.04705941702553	-1.95490106992737
C 3.24203367999152	7.28423445956157	-1.16583093961863
H 3.56832550317920	6.28884447976334	-1.50465634412033
H 1.99426163265661	7.65195683021970	-2.91340929070970

C	3.71515564024440	7.78762389302061	0.05607578111698
H	4.41845075891517	7.18269828231720	0.64861725710564

Cartesian coordinates for **3a**

Fe	-4.28432384517194	-1.08635026421967	14.49345434516177
Fe	0.38110441495623	3.37136145016491	15.27134811087277
P	-1.13355655726442	3.45452891538698	13.52660563874998
P	-3.05768887263011	2.27039888607939	13.88994855536273
P	-0.95829653179676	2.20066717604805	11.64461124835378
P	-2.36918391933369	0.13730449711481	13.94839020816314
P	-2.87975820179563	1.05999448059073	11.94867595167144
O	-6.27399752683393	0.40835093726180	12.95957249644302
O	-0.99781271285914	1.27760176440884	16.75844564450651
O	2.06173263515945	1.33976589395275	14.01767481733544
O	-4.67283459117002	0.69395263594883	16.77667570397388
C	-4.18177097474932	-2.87553634228374	13.27790713855908
C	-3.08996392117691	-2.82874866382621	14.19910866801904
H	-2.03774584843683	-2.82615590930430	13.90324339716268
C	-4.13021573957701	-3.18603330464482	11.78305475353670
C	2.02255793369649	4.33819596293472	16.24558968966759
C	-3.54433914977195	-2.80769787722160	15.56712937306604
C	-1.30859706366093	3.31572893505732	10.22322462691571
C	0.86043185777133	4.27237742791809	17.09663473127102
H	0.82934695598464	3.75221809673559	18.05859490374191
C	0.46394538686825	1.17225448483395	11.11067755995518
C	0.24431378251242	5.51057592081118	15.29151364003471
H	-0.35633303753508	6.09871149580465	14.59203289929357
C	3.36848728659290	3.81126300974156	16.80450884575129
C	1.62086960839630	5.14916928377919	15.06983973148482
C	-5.15922971685563	-2.35606065429385	10.98686215686806
H	-6.18825070198836	-2.46560589717837	11.38360017285219
H	-5.18194235243724	-2.69234512203073	9.93040705602872
H	-4.90271646004744	-1.27840562343823	10.99192508643703
C	-5.36051322449343	-2.83853738626779	14.10226446099879
H	-6.38504099434870	-2.86158010425530	13.71633388778511
C	-0.24506210829508	5.00615136728649	16.53452624094781
C	-5.02699965391393	-2.80956898348829	15.50457337417935
C	2.40577046111582	5.73944358679540	13.87349273054795

C -2.53261106943822	-2.96778415785412	16.73163965445164
C -5.44104056881643	-0.16133314783903	13.53923532268880
C 1.43218854026837	1.70350507668751	10.22603794548602
H 1.31712294167105	2.71865919578743	9.81913894789621
C -2.72098151694810	-2.94001837297040	11.20998645936072
H -2.40907671200388	-1.88489823508430	11.34402268114765
H -2.70864805196560	-3.15775758063176	10.12312238862765
H -1.95845249141404	-3.59275268583833	11.68126957728109
C 4.45908638776180	3.46368643182441	15.77100369179582
H 4.13617656841163	2.66829719626300	15.07297578806444
H 5.35022874586805	3.08141732040828	16.30803445129287
H 4.79611542363540	4.33490847855530	15.18237214914751
C 1.72045930760550	-0.91221990999457	11.22593546402446
H 1.83751405856225	-1.93203605510205	11.62136837827411
C 1.39456036388918	2.17934139679477	14.47081251485488
C 0.62173293647050	-0.13481290916619	11.62225327666467
H -0.11054685878318	-0.53097434401524	12.34307592255851
C -4.49187864473015	-0.02070226765552	15.87569509316664
C 3.55778496927620	6.62685775413403	14.41339767428180
H 3.17647426321698	7.39907518906846	15.11175669587889
H 4.05108945699246	7.15260857687259	13.57090832438602
H 4.33815246176344	6.04874659996578	14.94060101904093
C 3.13853868889570	2.52990453128964	17.64734814542216
H 2.50973409484967	2.70095390122969	18.54225212280535
H 4.11363931894377	2.15767489734551	18.01888757843645
H 2.67708133066863	1.72006610958274	17.04835078615872
C 2.94853336627422	4.66667520968902	12.90201138644809
H 3.59231111843788	3.91797947985272	13.39044667197397
H 3.54574786105125	5.15104102239227	12.10256475748594
H 2.11371037834853	4.12432376412923	12.41492116184816
C 2.67555975430061	-0.38762016079618	10.33818562684948
H 3.53796181413491	-0.99884256114320	10.03312200652596
C -6.16535664252833	-3.00655976235871	16.53583839067441
C -1.57742979756871	4.68863882360059	10.39238750917242
H -1.56644069692265	5.12231261920019	11.40420795148386
C -1.37543048263854	2.74127828311424	8.93106285543512
H -1.18523825534704	1.66642444884602	8.78707251417854
C -4.47485580150624	-4.69447055192677	11.64477780543668

H -3.77184113085546	-5.32663508155447	12.22389116080596
H -4.41328277577888	-5.00385573792441	10.58134722198741
H -5.50150922476166	-4.91428351890760	12.00060883634232
C 3.90498552407290	4.92001439879648	17.75377314146854
H 4.11323020912858	5.86487381853761	17.21631537042123
H 4.85004634711436	4.58548054034794	18.22791285029179
H 3.18207709684721	5.14557134445537	18.56329829377528
C 2.53199434142094	0.91997758702134	9.84392397600968
H 3.27836524483485	1.33379160463648	9.14966273698435
C -1.68804739678141	3.54370840219907	7.82437774627766
H -1.73817327076851	3.09226964582122	6.82240284137163
C -1.93610423080970	4.91796786373506	7.99412957376906
H -2.18339276707158	5.54332018591288	7.12340125685244
C -1.88252041988417	5.48696695985515	9.27661231612985
H -2.09003807923038	6.55833096023456	9.41530218213261
C -1.56077062410225	5.38026237299359	17.21504079605489
C -1.39769315739872	6.84882409956995	17.69651660054159
H -0.54275390819908	6.95498355977341	18.39442523278737
H -2.31257741160858	7.17844164767431	18.22944862822595
H -1.23208913196109	7.54223315970254	16.84756395362416
C -0.48993335573157	2.11140536134737	16.12463336320094
C 1.48759735295817	6.66503201159138	13.03826494857312
H 0.61587669250874	6.12078930441512	12.62204315468960
H 2.06083949861827	7.06810155902522	12.18022125093042
H 1.11740351927402	7.53344133640575	13.61881266211213
C -2.74673538138279	5.29551751754009	16.23142549209401
H -2.59396371716008	5.91943999498399	15.32791448007250
H -3.67552981834100	5.65155938293559	16.72085471526922
H -2.92591542938196	4.25221377283484	15.89929754071711
C -1.84154153073634	4.48171826454922	18.43680949975048
H -1.93380873571883	3.41392051213543	18.15853379225103
H -2.79687347151602	4.78130655188271	18.91213653991218
H -1.05405472003849	4.57294430297805	19.21207779289810
C -6.46969366827795	-4.53073652233897	16.56558196047142
H -6.74363512293890	-4.90977603163459	15.56064149057548
H -7.32168762513735	-4.73298045559899	17.24638958728093
H -5.60379531132705	-5.12034680466513	16.92367044041973
C -1.08453755592253	-3.00811992898669	16.18091837437208

H -0.90687842817235	-3.87375990754388	15.51174229997505
H -0.37749224466553	-3.10506022614956	17.02867103891087
H -0.81918182602333	-2.07753597270808	15.63899903454442
C -2.79125666715952	-4.33688330052161	17.41500179998911
H -3.76842979125112	-4.38166930640628	17.92989188453145
H -2.00849550692229	-4.52977758928258	18.17669303364558
H -2.75643537991244	-5.16632547198252	16.67994648110616
C -2.56990692180060	-1.82304864165864	17.76789297508499
H -2.23809932337913	-0.86878908317225	17.31454467576807
H -1.87291178836409	-2.05396864513687	18.59939354535644
H -3.56613573064386	-1.66000827521313	18.20821981681405
C -5.88099721007206	-2.52561881716565	17.97297537231664
H -5.04856055227088	-3.06735921135310	18.45640997782057
H -6.78041361409629	-2.70719017412157	18.59463816438281
H -5.66993880131754	-1.44028277637191	18.01648749875545
C -7.44684556664476	-2.26618524498277	16.07135481438912
H -7.27616547534453	-1.17581244994261	15.97339855449829
H -8.24711550060002	-2.41416046920333	16.82299514171477
H -7.84795970291251	-2.64092205982401	15.10993355254547

Cartesian coordinates for **3a**−CO

Fe -3.79274893401707	-0.43117540822468	14.69834596898268
Fe -0.16455514756921	2.47425853107216	14.89366228948742
P -0.96542257714830	3.78192660908364	13.20257730288496
P -3.12257926449535	2.82785801719101	13.14670551826543
P -0.87931978048603	2.61232248399241	11.29839357117721
P -2.05239036926348	1.20438391724470	14.23955610715702
P -2.28191883678942	1.00628962439929	11.98740613479659
O -5.63284041201494	0.35637800382934	12.57169963888122
O 1.67086463668876	0.81877080682433	13.37577892303305
O -5.23933415717160	1.58853112335828	16.21170732553819
C -3.57056809101099	-2.53671063888149	14.00843027225371
C -2.45273861146942	-2.22137205537041	14.89507378642024
C 0.21909088640953	2.25315971203630	16.96135153919878
C -3.03283929015984	-1.77623547876756	16.14900908840967
C -1.76683375683805	3.55450919432219	9.99696573086388
C -0.80745417193434	3.25271832458963	16.85504662748795

C 0.65908138889822	2.03252467024500	10.50279712900297
C 1.11617609949291	4.00417201083299	15.68992557714011
C 1.42942049605003	2.69998121263024	16.30967212395317
C -4.77848274192223	-2.27910778701995	14.77997454144299
C -0.26193997808202	4.28401711240403	16.04369428311523
C -4.46513672706116	-1.89493390331271	16.11798191555630
C -4.80352807698086	0.06612082185305	13.33503221559349
C 1.54691924527975	2.98180361112802	9.94592836435172
H 1.32244640877816	4.05842583241473	10.00692929045469
C 2.10441758997176	0.22720775798698	9.74401753516963
H 2.32061810237286	-0.84879563016811	9.66065115688307
C 0.90265331050501	1.48001784357785	13.96657425025658
C 0.94168557164806	0.65505766702732	10.40454024815515
H 0.25524970959564	-0.08505623201186	10.84542095888627
C -4.59709176835826	0.79708787950273	15.64767147721786
C 2.99142326707553	1.17000844069731	9.19769507500065
H 3.90550833497280	0.83114184159938	8.68673341774186
C -2.30168688841297	4.83505033936373	10.25331558532064
H -2.16273626263765	5.30451064143123	11.24087673497659
C -1.92680115034937	2.96383578995411	8.72209421372581
H -1.50514101711237	1.96899372088574	8.51061621024881
C 2.71406311040656	2.54545712828857	9.30145838103545
H 3.40752578727893	3.28347334278537	8.87084727753810
C -2.62267583091782	3.65248849450882	7.71764293108936
H -2.74827737594602	3.18961156497411	6.72700452959975
C -3.15502785693372	4.92879416478582	7.97376578917213
H -3.69627237978532	5.46712161263052	7.18086334570149
C -2.99120198158756	5.51873221939898	9.23805619164085
H -3.39817194377432	6.52105462428302	9.43841673191512
H 0.13324337664713	1.32670010778166	17.53659265064532
H -0.79598263617078	5.19907143242913	15.76857356155015
H -2.45881878074236	-1.47833268920434	17.02983767575366
H -5.79396133586183	-2.44935613423727	14.40754664817624
C -5.42902995478625	-1.91167120197980	17.30414862822415
C -5.49310377209706	-3.40658656948586	17.73768846048051
H -5.87644646107529	-4.05138024410736	16.92118435061351
H -6.17353854688440	-3.51444879618793	18.60701308844901
H -4.49519130124577	-3.78714755654752	18.03516117215141

C -6.84888921565381	-1.45002800429743	16.90698249823079
H -7.27244223423443	-2.06412335265014	16.08682673580662
H -7.53105070803927	-1.55544452796952	17.77433691284298
H -6.86774366867830	-0.38860105608842	16.59369409206458
C -4.91519091242102	-1.07749002090686	18.49502564692337
H -5.61729456864227	-1.17257246285150	19.34803941509978
H -3.92576612335808	-1.43006230684773	18.85062594990987
H -4.83562346848133	-0.00171700977011	18.24951158902283
C -3.73290612639183	-3.32213094593134	12.68014456392559
C -4.01990515632537	-4.79190837443552	13.11148163286516
H -3.18802495210261	-5.21982894291296	13.70391029928859
H -4.15950858023284	-5.42478148301176	12.21146413024788
H -4.94225626697614	-4.86325929241185	13.72260878871443
C -2.53668233178256	-3.31263115905206	11.71212140775382
H -1.65059533607957	-3.83076281515055	12.12016244062459
H -2.82183626476124	-3.85080103205428	10.78589509196604
H -2.25478951977403	-2.28144362724667	11.42214042967373
C -4.94766195438659	-2.82107296440243	11.85745728242455
H -5.10806103558575	-3.51039481318067	11.00497196184321
H -5.89699476565693	-2.79646327555904	12.42630817092031
H -4.76708743298938	-1.81351064291502	11.43836079372020
C -0.93366038807072	-2.50594849111312	14.81261661860111
C -0.21900501822698	-1.93248754071057	16.06035836311983
H -0.34630003359766	-0.83262242963649	16.12796722570407
H 0.86614369796847	-2.14043836421768	15.98324824361138
H -0.56930286284660	-2.39432418798939	17.00453492631174
C -0.21866562440198	-1.90132189710108	13.58682567240050
H -0.37294621112859	-0.80769611716316	13.54614569069479
H 0.87224851474041	-2.08200554043651	13.66631580911454
H -0.55753376842338	-2.32870766442255	12.63192597905620
C -0.73998874205827	-4.04589104758989	14.83977776823799
H 0.34236577900258	-4.28172335193825	14.90246350174265
H -1.23611764685925	-4.50291197102140	15.72029226527273
H -1.13336667879232	-4.53660018899378	13.93025401390074
C 1.98972829275856	5.08245427592940	14.99554081513650
C 3.11083624819714	5.55387174517046	15.96045693112976
H 2.68819172700955	5.88895506839561	16.92966698931976
H 3.64304516080822	6.41839969672773	15.51309255486704

H 3.86631805912720	4.77541730336186	16.16936553689704
C 1.14387610598092	6.34466438060886	14.68318032577837
H 0.75022318984578	6.82276283627818	15.60288176848022
H 1.78499998390645	7.09309207163459	14.17542575453169
H 0.29408264411178	6.12249378352266	14.00751714525605
C 2.59038866800226	4.60186314976150	13.65304516397056
H 3.25702804720448	5.38390081222246	13.23365283484870
H 3.17433031203279	3.67133696178964	13.74987027630929
H 1.78411141756165	4.41182662989516	12.91541482320754
C 2.75593455237989	1.95841352760666	16.62113197267238
C 2.51538606469149	0.44130081728962	16.82034512946971
H 1.82114716722978	0.21848897648801	17.65428271262075
H 3.47790335761163	-0.04822158551255	17.07069922468538
H 2.12763489698683	-0.03580976404868	15.90105509218789
C 3.23965795903209	2.53377626532860	17.98585693798961
H 2.48032533965027	2.37354192182585	18.77793446857617
H 4.17267838283447	2.02157020735630	18.29800068719972
H 3.44707635005361	3.61886084698327	17.93177514670373
C 3.89492176470733	2.08637012171174	15.58758726714621
H 4.77400024151341	1.52257810260204	15.95963067893735
H 3.61862036623567	1.65433821429396	14.60747762425740
H 4.22670769354692	3.12668726365280	15.43286338025723
C -2.08526857005474	3.35373288368588	17.67751075570891
C -1.73448261055044	4.21875910017361	18.92300194868951
H -0.91205106121377	3.76198008723811	19.51032197043137
H -2.62145332248529	4.30876502473551	19.58412688990003
H -1.42111243795459	5.24164926004560	18.63197781358327
C -2.53482984763037	1.96347960145948	18.16828827438465
H -1.79103933044759	1.51990244014614	18.86162552166951
H -3.49261057090050	2.04301125659659	18.71944769956563
H -2.67841594117193	1.25733817432605	17.32627515054448
C -3.20739334667797	4.05865988849017	16.88758921489974
H -4.13245733245737	4.11682272655561	17.49512155905033
H -2.92438257816234	5.09588479834239	16.61881674162069
H -3.45493659104368	3.52156012147193	15.95036576310923

Cartesian coordinates for $[Ph_2P]^+$

H -2.81029505290288	6.18397711900421	9.73143037677821

C -2.24702713037520	5.27392744734249	9.47754953187262
C -1.90202971837021	4.36151777232332	10.47640779071743
H -2.20281528533194	4.54836769972163	11.52029975337520
C -1.13713761431867	3.18937994572326	10.15672250278754
C -0.80527590809520	2.92891836861653	8.78789233436006
P -0.81735175870561	2.14943739386979	11.56292238883900
H 1.62219666368201	2.25248355329552	9.65428430310182
C 1.60851311628775	1.32501557616092	10.24426236489007
C 0.56035497790163	1.08868204700339	11.19123147693557
C 2.65309650238373	0.40938938836373	10.11992226993722
C 0.63139102278270	-0.07217765212211	12.03285928986797
C 2.67353777695092	-0.75773397638718	10.91838497280118
H -0.14587056829463	-0.24034308566063	12.79655356104819
C 1.66428772162779	-0.99835467523601	11.87317508848743
H 1.69999374350247	-1.90093365438379	12.50112644638162
H -2.18521851750466	5.71609500237519	7.34797467426603
C -1.88969314153858	5.00991548890581	8.13945318165062
C -1.18553003501509	3.83235768468136	7.79603181936613
H -0.95144011831427	3.62165862347850	6.74170058125130
H -0.30081549306715	1.99334005582346	8.50744766779533
H 3.47242565119205	0.60217735761383	9.41088051151404
H 3.50320316552303	-1.47409748051324	10.81018711197529

Appendix

List of Figures

S1 $^{31}P\{^1H\}$ VT NMR spectrum from the reaction of **1** with $[Ph_2P][OTf]$ 4
S2 $^{31}P\{^1H\}$ NMR spectrum from the reaction of **1** with $[Ph_2P][OTf]$ 5
S3 $^{31}P\{^1H\}$ NMR spectrum from the reaction of 1 with $[Ph_2P][OTf]$. 6
S4 ^{31}P-^{31}P COSY NMR spectrum of **2a** and **3a** at 193 K 7
S5 Simulation of the spin systems in the $^{31}P\{^1H\}$ NMR spectrum of compounds **2a** and **3a**. 8
S6 $^{31}P\{^1H\}$ VT NMR spectrum of the reaction of 1 with $[Me_2P][OTf]$. 9
S7 $^{31}P\{^1H\}$ NMR spectrum from the reaction of **1** with $[Me_2P][OTf$. 10
S8 ^{31}P-^{31}P COSY NMR spectrum of 2b and 3b at 193 K 11

S9 Simulation of the spin systems in the $^{31}P\{^{1}H\}$ NMR spectrum of compounds **2b** and **3b** 12
S10 Simulation (inverted) of the $^{31}\{^{1}H\}$ NMR spectrum of **4**. 13
S11 $^{31}P\{^{1}H\}$ NMR spectrum from the reaction of **1** with $[Ph_2P][PF_6]$. 14
S12 ^{31}P ^{31}P COSY NMR spectrum of **4** at room temperature. 15
S13 Simulation of the $^{31}P\{^{1}H\}$ NMR spectrum of coordination mode 1 and 2 of $[Ph_2As][OTf]$ on **1**. 16
S14 Comparison of the $^{31}P\{^{1}H\}$ NMR spectrum of coordination mode 1 and 2 of $[Ph_2As][OTf]$ on **1** at 193 K and room temperature. 17
S15 $^{31}P\{^{1}H\}$ NMR spectrum from the reaction of **1** with $[Ph_2As][OTf]$. 18
S16 ^{31}P ^{31}P COSY NMR spectrum of **5** at 273 K. 19
S17 ^{31}P VT NMR spectrum of **5**. 20
S18 ^{31}P NMR spectrum of **5**. 21
S19 ^{1}H NMR spectrum of **5** in CD_2Cl_2 at room temperature. 22
S20 Molecular structure of **3a** in the solid state. 24
S21 Molecular structure of **4** in the solid state. 25
S22 Molecular structure of **5** in the solid state. 26

List of Tables

S1 $^{31}P\{^{1}H\}$ NMR data of **2a**: (CD_2Cl_2; 193 K): 2
S2 $^{31}P\{^{1}H\}$ NMR data of **3a**: (CD_2Cl_2; 193 K): 2
S3 $^{31}P\{^{1}H\}$ NMR data of **2b**: (CD_2Cl_2; 193 K): 2
S4 $^{31}P\{^{1}H\}$ NMR data of **3b**: (CD_2Cl_2; 193 K): 3
S5 $^{31}P\{^{1}H\}$ NMR data of **4**: (CD_2Cl_2; 300 K): 3
S6 $^{31}P\{^{1}H\}$ NMR data of coordination mode 1 of $[Ph_2As][OTf]$ on **1** from the simulation (in CD_2Cl_2 at 193 K). 3
S7 $^{31}P\{^{1}H\}$ NMR data of coordination mode 2 of $[Ph_2As][OTf]$ on **1** from the simulation (in CD_2Cl_2 at 193 K). 3
S8 ^{31}P NMR data of **5**: (CD_2Cl_2; 273 K):. 18
S9 Crystallographic data for **3a**, **4** and **5**: 23
S10 Thermodynamic data of **1**, **2a**, **3a**, the decarbonlyated form of **3a**, **4**, CO and $[Ph_2P]^+$ 27

References

[1] Sheldrick, G.M. *SHELXT* – Integrated space-group and crystal-structure determination. *Acta Crystallogr., Sect. A* 2015. *71*, 3–8. doi: 10.1107/S2053273314026370

[2] Dolomanov, O.V.; Bourhis, L.J.; Gildea, R.J.; Howard, J.A.K.; and Puschmann, H. OLEX2: a complete structure solution, refinement and analysis program. *J. Appl. Crystallogr.* 2009. *42*, 339–341. doi: 10.1107/S0021889808042726

[3] Sheldrick, G.M. Crystal structure refinement with *SHELXL*. *Acta Crystallogr., Sect. C* 2015. *71*, 3–8. doi:10.1107/S2053229614024218

[4] Burkhardt, A.; Pakendorf, T.; Reime, B.; Meyer, J.; Fischer, P.; Stübe, N.; Panneerselvam, S.; Lorbeer, O.; Stachnik, K.; Warmer, M.; Rödig, P.; Göries, D.; and Meents, A. Status of the crystallography beamlines at PETRA III. . *Eur. Phys. J. Plus* 2016. *131*, 56–64. doi:10.1140/epjp/i2016-16056-0

[5] Neese, F.; Wennmohs, F.; Becker, U.; and Riplinger, C. The ORCA quantum chemistry program package. *J. Chem. Phys.* 2020. *152*, 224108. doi: 10.1063/5.0004608

[6] Becke, A.D. Density-functional exchange-energy approximation with correct asymptotic behavior. *Phys. Rev. A* 1988. *38*, 3098–3100. doi: 10.1103/PhysRevA.38.3098

[7] Perdew, J.P. Density-functional approximation for the correlation energy of the inhomogeneous electron gas. *Phys. Rev. B* 1986. *33*, 8822–8824. doi:10.1103/PhysRevB.33.8822

[8] Perdew, J.P. Erratum: Density-functional approximation for the correlation energy of the inhomogeneous electron gas. *Phys. Rev. B* 1986. *34*, 7406–7406. doi:10.1103/PhysRevB.34.7406

[9] Weigend, F. and Ahlrichs, R. Balanced basis sets of split valence, triple zeta valence and quadruple zeta valence quality for H to Rn: Design and assessment of accuracy. *Phys. Chem. Chem. Phys.* 2005. *7*, 3297–3305. doi:10.1039/B508541A

[10] Weigend, F. Accurate Coulomb-fitting basis sets for H to Rn. *Phys. Chem. Chem. Phys.* 2006. *8*, 1057–1065. doi:10.1039/B515623H

[11] Hanwell, M.D.; Curtis, D.E.; Lonie, D.C.; Vandermeersch, T.; Zurek, E.; and Hutchison, G.R. Avogadro: an advanced semantic chemical editor, visualization, and analysis platform. *J. Cheminformatics* 2012. *4*, 17. doi: 10.1186/1758-2946-4-17
